# Analysis of the ArcA regulon in anaerobically grown *Salmonella enterica sv*. Typhimurium

**DOI:** 10.1186/1471-2180-11-58

**Published:** 2011-03-21

**Authors:** Matthew R Evans, Ryan C Fink, Andres Vazquez-Torres, Steffen Porwollik, Jessica Jones-Carson, Michael McClelland, Hosni M Hassan

**Affiliations:** 1 Department of Microbiology, North Carolina State University, Raleigh, North Carolina 27695-7615 USA; 2 Department of Microbiology, University of Colorado School of Medicine, Denver, Colorado 80262 USA; 3 The Vaccine Research Institute of San Diego, 10835 Road to the Cure, Suite 105, San Diego, California 92121 USA; 4 Pfizer, Inc., 4300 Oak Park Road, Sanford, NC 27330-9550 USA; 5 Department of Food Science and Nutrition, University of Minnesota, St. Paul, MN 55108-1038 USA

## Abstract

**Background:**

*Salmonella enterica *serovar Typhimurium (*S*. Typhimurium) is a Gram-negative pathogen that must successfully adapt to the broad fluctuations in the concentration of dissolved dioxygen encountered in the host. In *Escherichia coli*, ArcA (Aerobic Respiratory Control) helps the cells to sense and respond to the presence of dioxygen. The global role of ArcA in *E. coli *is well characterized; however, little is known about its role in anaerobically grown *S*. Typhimurium.

**Results:**

We compared the transcriptional profiles of the virulent wild-type (WT) strain (ATCC 14028s) and its isogenic *arcA *mutant grown under anaerobic conditions. We found that ArcA directly or indirectly regulates 392 genes (8.5% of the genome); of these, 138 genes are poorly characterized. Regulation by ArcA in *S*. Typhimurium is similar, but distinct from that in *E*. *coli*. Thus, genes/operons involved in core metabolic pathways (e.g., succinyl-CoA, fatty acid degradation, cytochrome oxidase complexes, flagellar biosynthesis, motility, and chemotaxis) were regulated similarly in the two organisms. However, genes/operons present in both organisms, but regulated differently by ArcA in *S*. Typhimurium included those coding for ethanolamine utilization, lactate transport and metabolism, and succinate dehydrogenases. *Salmonella*-specific genes/operons regulated by ArcA included those required for propanediol utilization, flagellar genes (*mcpAC*, *cheV*), Gifsy-1 prophage genes, and three SPI-3 genes (*mgtBC*, *slsA*, STM3784). In agreement with our microarray data, the *arcA *mutant was non-motile, lacked flagella, and was as virulent in mice as the WT. Additionally, we identified a set of 120 genes whose regulation was shared with the anaerobic redox regulator, Fnr.

**Conclusion(s):**

We have identified the ArcA regulon in anaerobically grown *S*. Typhimurium. Our results demonstrated that in *S*. Typhimurium, ArcA serves as a transcriptional regulator coordinating cellular metabolism, flagella biosynthesis, and motility. Furthermore, ArcA and Fnr share in the regulation of 120 *S*. Typhimurium genes.

## Background

*Salmonella enterica *serovar Typhimurium (*S*. Typhimurium) is a Gram-negative intracellular pathogen that causes gastroenteritis in the human host. Although non life-threatening in healthy adults, it can be fatal for children and immunocompromised individuals. The infection proceeds via two main stages: invasion and systemic infection. During the invasion stage, the pathogen adheres and colonizes the intestines gaining access to the epithelial cells. Subsequently, *Salmonella *crosses the epithelial cells and gets internalized by the macrophages where it reproduces and stealthily spreads in the host and causes systemic infection [1-4]. Clearly, *Salmonella *must adapt quickly to the diverse environments it encounters. In fact, from the gastrointestinal tract to the intracellular milieu, it is challenged with fluctuations in oxygen concentration and with numerous host-immune defenses including a battery of reactive oxygen (ROS) and nitrogen species (RNS) and antimicrobial peptides that reduce its ability to colonize the host [1-4].

In *Escherichia coli*, ArcA (Aerobic Respiratory Control) is one of the main transcriptional regulators involved in the metabolic shift from anaerobic to aerobic conditions and controlling the enzymatic defenses of bacteria against ROS. ArcA is a cytosolic response regulator of a two-component global regulatory system, ArcA/ArcB, where ArcB is a transmembrane histidine kinase sensor. ArcB transfers a phosphoryl group to ArcA, activating it, and inducing or repressing a large and diverse number of operons including the tricarboxylic acid cycle (TCA), terminal oxidases, dehydrogenases of the flavoprotein class, the glyoxylate shunt, and fatty acid degradation [5-17]. Due to its importance in diverse energy metabolic processes, the ArcA regulon has been thoroughly characterized in *E. coli *[5,12,18]. Conversely, very little is known about the regulatory network controlled by ArcA in *S*. Typhimurium under anaerobic conditions.

Although *E*. *coli *and *S*. Typhimurium share a very high genomic similarity (~75-80%) [19], we previously discovered that the Fnr (Fumarate Nitrate Reductase) regulon of *S*. Typhimurium is markedly different from the one identified in *E*. *coli *[20]. Due to the complementary roles of ArcA and Fnr in the regulation of cellular metabolism and adaptation to changes in redox, we hypothesized that the ArcA regulon of *S*. Typhimurium will also differ from that of *E. coli*. The results indicate that in *S*. Typhimurium, as in *E*. *coli*, the ArcA regulon includes the core metabolic and energy functions as well as motility. However, *Salmonella*-specific genes/operons regulated by ArcA include newly identified flagellar genes (*mcpAC*, *cheV*), Gifsy-1 prophage genes, a few SPI-3 genes (*mgtBC*, *slsA*, STM3784), and those for propanediol utilization. Furthermore, the *arcA *mutant was non-motile and was as virulent as the isogenic wild-type strain. We also identified 120 genes that were regulated by the anaerobic regulator, Fnr, as well as by ArcA.

## Methods

### Bacterial strains and growth conditions

The bacterial strains used in this study are listed in Table 1. Wild-type (WT) *S*. Typhimurium (14028s) and its isogenic *arcA *mutant (NC 980) were used throughout. P22 phage was used to move the *arcA*::Tn*10 *mutation from *S*. Typhimurium LT2 (TT17442) [21] to strain 14028 s. Transductants were plated on Evans Blue Uranine (EBU) agar and the *arcA *mutant was tested for its inability to grow on toluidine blue agar [22]. The Tn*10 *insertion junctions of the *arcA *mutant were confirmed by PCR and DNA sequencing. Additionally, the absence of the ArcA protein in the mutant was confirmed by Western blotting (Additional file 1: Figure S1 - lane 3).

**Table 1:** List of strains, plasmids, and phage used in this study

Strain, Plasmid, or Phage	Relevant Characteristics	Source and/or Reference
**Strains**		

*Salmonella *Typhimurium		
14028s	Wild-type	American Type Culture Collection
TT17442/SL3052	(LT2) containing *metE205 ara*-*9 cob*-*24*::MudJ *arcA201*::Tn*10d*-Tet	[21/S. Libby]
		
NC980	14028 s containing *arcA*::Tn*10 *(Tet^r^) [TT17442 (P_22_) × 14028s]	This study

NC989	Same as NC980, but harboring p*arcA*.	This study

*Escherichia coli*		
ER2420	Harboring pACYC177	New England Biolabs
		
ER2925	*ara-14 leuB6 fhuA31 lacY1 tsx78 glnV44 galK2 galT22 mcrA dcm-6 hisG4 rfbD1 R(zgb210*::Tn*10)TetS endA1 rpsL136 dam13*::Tn*9 xylA-5 mtl-1 thi-1 mcrB1 hsdR2*	New England Biolabs

**Plasmids**		

pACYC177	F^- ^*ara-14 leu fhuA2 *Δ*(gpt-proA)62 lacY1 glnV44 galK2 rpsL20 xyl-5 mtl-1 *Δ*(mcrC-mrr)*_HB101_	New England Biolabs
p*arcA*	An 897 base pair *arcA *amplicon from *S*. Typhimurium 14028 s cloned into the *Sma*I site within the Kan^r ^gene of pACYC177.	This study

**Phage**		

P22		S. Libby/Collection stock of NC

Unless stated otherwise, the WT and the *arcA *mutant were grown anaerobically at 37°C in MOPS-buffered (100 mM, pH 7.4) LB broth supplemented with 20 mM D-xylose (LB-MOPS-X). MOPS was used in the medium to avoid the indirect effects of pH, while xylose was used to avoid the effects of catabolite repression [12]. An anaerobic chamber (Coy, Ann Harbor, MI) with anaerobic gas mixture (10% H_2_, 5% CO_2_, and 85% N_2_) was used as previously described [20]. All solutions were anaerobically pre-equilibrated in the chamber for 48 h before use. Overnight cultures (15-18 h) were used to inoculate fresh media. Aerobic growth was carried-out using LB or LB-MOPS-X as specified (volume of the culture: flask ratio = 1:5, shaking at 200 rpm using an orbital shaker). Growth kinetic experiments were performed on the WT and the *arcA *mutant in triplicate under both aerobic and anaerobic conditions.

### Construction of p*arcA*

For complementation studies, a low-copy-number plasmid, expressing *arcA *(p*arcA*, NC 989) was constructed. The complete *arcA *sequence starting from 180 bp upstream from the start codon (ATG) until the stop codon (TAA) of *arcA *[i.e., 897 bp fragment] was amplified from the WT strain using the following primers (Integrated DNA Technologies, Coralville, IA):

*arcA*-Forward 5'- TCGATCCCGGGTACCCACGACCAAGCTAATG-3' and *arcA*-Reverse 5'-CTACCTCCCGGGTTAATCCTGCAGGTCGCCG -3' [*Sma*I site underlined]. The PCR product was digested with *Sma*I and ligated into the pACYC177 (New England BioLabs, Ipswich, MA) vector that was also cut with *Sma*I. Thus, in the new plasmid (p*arcA*) the Kan^r ^gene in pACYC177 was disrupted by the insertion of *arcA*. Plasmid DNA (p*arcA*) was first transformed into a restriction deficient strain of *E*. *coli *[ER2925 (New England BioLabs)], which was subsequently purified and transformed into and maintained in the *S*. Typhimurium *arcA *mutant, thus generating NC989 (Table 1).

Transformations were carried-out using the calcium chloride method [23]. Plasmid DNA and genomic DNA were isolated using the Qiagen Mini Spin isolation kit (Qiagen, Valencia, CA) and the DNAeasy Tissue Kit (Qiagen), respectively. Transformants containing p*arcA *(NC989) were confirmed for Amp^r ^(130 μg/ml) and Kan^s ^(50 μg/ml) on LB plates and the presence of p*arcA *was confirmed via PCR and restriction analysis. The expression of ArcA was confirmed by Western blot analysis (Additional file 1: Figure S1 - lane 4).

### RNA isolation

Overnight anaerobic cultures of the WT or the *arcA *mutant were used to inoculate three independent flasks for each strain. Every flask contained 150 ml of LB-MOPS-X equilibrated in the anaerobic gas mix for the previous 48 h. The three independent cultures of each strain were grown to an OD_600 _= 0.30-0.35, pooled, and treated with RNAlater (Qiagen, Valencia, CA) to fix the cells and preserve the quality of the RNA. Total RNA was extracted using RNeasy RNA extraction kit (Qiagen) and the samples were treated with RNase-free DNase (Invitrogen, Carlsbad, CA). The absence of contaminating DNA and the quality of the RNA was confirmed by the lack of PCR amplification of known genes (i.e.: *fnr*) and by using agarose-gel electrophoresis. Aliquots of the RNA samples were kept at -80°C for use in the microarray and the qRT-PCR studies.

### Microarray studies

*S*. Typhimurium microarray slides were prepared and used as previously described [24]. For the hybridizations, the SuperScript™ Indirect cDNA Labeling System (Invitrogen) was used to synthesize the cDNA from the RNA prepared from the WT and *arcA *mutant strains. Dye swapping was performed to avoid dye-associated effects on cDNA synthesis. Slide hybridizations and scanning were carried-out using the same protocols and equipment as previously described [20].

### Data analysis

Cy3 and Cy5 values for each spot were normalized over the total intensity for each dye, to account for differences in total intensity between the two scanned images. The consistency of the data obtained from the microarray analysis was evaluated using two methods: (i) a pair-wise comparison, calculated with a two-tailed Student's *t *test and analyzed by the MEAN and TTEST procedures of SAS-STAT statistical software (SAS Institute, Cary, NC) [the effective degrees of freedom for the *t *test were calculated as previously described [25]; and (ii) a regularized *t *test followed by a posterior probability of differential expression [PPDE (p)] method. The signal intensity at each spot from the *arcA *mutant and the WT were background-subtracted, normalized, and used to calculate the ratio of gene expression between the two strains. All replicas were combined and the median expression ratio and standard deviations calculated for ORFs showing ≥ 2.5-fold change.

### Microarray data

The microarray data are accessible via GEO Accession Number GSE24564 at http://www.ncbi.nlm.nih.gov/geo/query/acc.cgi?acc=GSE24564.

### qRT-PCR

qRT-PCR [26] was used to validate the microarray data [27]. Seventeen genes were randomly chosen (Table 2) from the differentially expressed genes. Primers (Integrated DNA Technologies, Coralville, IA) were designed and qRT-PCRs were carried-out using QuantiTectTM SYBR^® ^Green RT-PCR Kit (Qiagen), an iCycler™ (Bio-Rad, Hercules, CA), and the data were analyzed by the Bio-Rad Optical System Software - Version 3.1, according to the manufacturer specifications. The cycling conditions comprised 30 min of a reverse transcriptase reaction at 50°C, 15 min of polymerase inactivation at 95°C, and 40 cycles each of 94°C for 15 sec for melting, 51°C for 30 sec for annealing, and 72°C for 30 sec for extension followed by 31 cycles each at 65°C for 10 sec to obtain the melt curve. To ensure accurate quantification of the mRNA levels, three amplifications of each gene were made using 1:5:25 dilutions of the total RNA. Measured mRNA levels were normalized to the mRNA levels of the housekeeping gene, *rpoD *(σ^70^). Normalized values were used to calculate the ratios of the expression levels [20] in the *arcA *mutant relative to the WT.

**Table 2:** Validation of microarray data using qRT-PCR of randomly selected genes relative to the housekeeping gene, *rpoD*^a^

**Locus**^**b**^	**Name**^**c**^	**Primer sequence**^**d**^	**Fragment (bp)**^**e**^	**Serovar Typhimurium Gene Function**^**f**^	Ratio of *arcA *mutant/WT		**Log**_**2 **_ratio	
					
					**qRT-PCR**^**g**^	**Microarray**^**h**^	**qRT-PCR**^**i**^	Microarray^j^
STM3217	*aer*	5'-CGTACAACATCTTAATCGTAGC-3' 5'-TTCGTTCAGATCATTATTACCC-3'	163	aerotaxis sensor receptor, senses cellular redox state or proton motive force	0.237	0.293	-2.1	-1.8

STM1919	*cheM*	5'-GCCAATTTCAAAAATATGACG-3' 5'-GTCCAGAAACTGAATAAGTTCG-3'	114	methyl accepting chemotaxis protein II, aspartate sensor-receptor	0.194	0.261	-2.4	-1.9

STM0441	*cyoC*	5'-TATTTAGCTCCATTACCTACGG-3' 5'-GGAATTCATAGAGTTCCATCC-3'	134	cytochrome o ubiquinol oxidase subunit III	4.920	5.465	2.3	2.5

STM1803	*dadA*	5'-TAACCTTTCGCTTTAATACTCC-3' 5'-GATATCAACAATGCCTTTAAGC-3'	155	D-amino acid dehydrogenase subunit	3.430	10.520	1.8	3.4

STM2892	*invJ*	5'-TTGCTATCGTCTAAAAATAGGC-3' 5'-TTGATATTATCGTCAGAGATTCC-3'	128	surface presentation of antigens; secretory proteins	0.855	1.010	-0.2	0.0

STM2324	*nuoF*	5'-GGATATCGAGACACTTGAGC-3' 5'-GATTAAATGGGTATTACTGAACG-3'	163	NADH dehydrogenase I chain F	0.380	1.706	-1.4	0.8

STM0650	STM0650	5'-CAACAGCTTATTGATTTAGTGG-3' 5'-CTAACGATTTTTCTTCAATGG-3'	130	putative hydrolase C-terminus	0.274	0.123	-1.9	-3.0

STM2787	STM2787	5'-AAGCGAATACAGCTATGAACC-3' 5'-ATTAGCTTTTGCAGAACATGG-3'	144	tricarboxylic transport	6.440	90.770	2.7	6.5

STM4463	STM4463	5'-AAGGTATCAGCCAGTCTACG-3' 5'-CGTATGGATAAGGATAAATTCG-3'	142	putative arginine repressor	0.165	0.012	-2.6	-6.4

STM2464	*eutN*	5'-AGGACAAATCGTATGTACCG-3' 5'-ACCAGCAGTACCCACTCTCC-3'	153	putative detox protein in ethanolamine utilization	0.181	0.159	-2.5	-2.7

STM2454	*eutR*	5'-GGTAAAAGAGCAGCATAAAGC-3' 5'-ATTATCACTCAAGACCTTACGC-3'	118	putative regulator ethanolamine operon (AraC/XylS family)	0.189	0.188	-2.4	-2.4

STM2470	*eutS*	5'-AATAAAGAACGCATTATTCAGG-3' 5'-GTTAAAGTCATAATGCCAATCG-3'	137	putative carboxysome structural protein, ethanol utilization	0.197	0.105	-2.3	-3.3

STM1172	*flgM*	5'-AGCGACATTAATATGGAACG-3' 5'-TTTACTCTGTAAGTAGCTCTGC-3'	126	anti-FliA (anti-sigma) factor; also known as RflB protein	0.196	0.163	-2.4	-2.6

STM3692	*lldP*	5'-TGATTAAACTCAAGCTGAAAGG-3' 5'-CCGAAATTTTATAGACAAAGACC-3'	189	LctP transporter, L-lactate permease	5.950	12.780	2.6	3.7

STM3693	*lldR*	5'-GAACAGAATATCGTGCAACC-3' 5'-GAGTCTGATTTTCTCTTTGTCG-3'	153	putative transcriptional regulator for lct operon (GntR family)	5.750	80.000	2.5	6.3

STM1923	*motA*	5'-GGTTATCGGTACAGTTTTCG-3' 5'-TAGATTTTGTGTATTTCGAACG-3'	194	proton conductor component of motor, torque generator	0.282	0.253	-1.8	-2.0

STM4277	*nrfA*	5'-GACTAACTCTCTGTCGAAAACC-3' 5'-ATTTTATGGTCGGTGTAGAGC-3'	159	nitrite reductase periplasmic cytochrome c(552)	0.314	0.285	-1.7	-1.8

^a^STM3211 (*rpoD*) is a housekeeping gene that was used as the reference gene where no significant

change in expression level was observed. The primer sequences (5' to 3') used for *rpoD *were as follows: CGATGTCTCTGAAGAAGTGC (forward) and TTCAACCATCTCTTTCTTCG (reverse). The size of the fragment generated is 150 bp.

^b^Location of the open reading frame (ORF) in the *S*. Typhimurium LT2 genome.

^c^Respective gene name or symbol.

^d^For each set, the first primer is the forward primer and the second primer is the reverse primer.

^e^Size of the amplified PCR product.

^f^Functional classification according to the KEGG (Kyoto Encyclopedia of Genes and Genomes) database.

^g^Expression levels of quantitative reverse transcriptase polymerase chain reaction - values shown as the ratio between the *arcA *mutant and the wild-type; where values <1 indicate that ArcA acts as an activator, and values >1 indicate ArcA acts as a repressor.

^h^Expression levels from the microarray data - values shown as the ratio between the *arcA *mutant and the wild-type; where values <1 indicate that ArcA acts as an activator, and values >1 indicate ArcA acts as a repressor.

^i^Expression levels of quantitative reverse transcriptase polymerase chain reaction comparing the *arcA *mutant versus the wild-type - shown in signal to log_2 _ratio (SLR).

^j^Expression levels of microarray data comparing the *arcA *mutant versus the wild-type - shown in signal to log_2 _ratio (SLR).

### Logo graph and promoter analysis

The information matrix for the generation of the ArcA logo was produced using the alignment of the *E. coli *ArcA binding sequences, available at http://arep.med.harvard.edu/ecoli_matrices/[28]. The alignment of the ArcA motifs from this website did not include the motifs present in the *sodA *and *mutM *promoters [29,30], therefore they were included in our analysis. To account for differences in nucleotide usage or slight variations in consensus sequences, a second alignment was built for *S*. Typhimurium using the 5'-regions of the homologous genes originally used to build the *E. coli *information matrix. The *Salmonella *alignment was used to prepare a new information matrice using the Patser software (version 3d), available at http://rsat.ulb.ac.be/rsat/[31] and graphed using the Weblogo software (version 2.8.1, 2004-10-18), available at http://weblogo.berkeley.edu/[32].

### Swarming motility assay and electron microscopy

The swarming of the WT and the *arcA *mutant were evaluated under anoxic conditions. Ten microliters of anaerobically grown cells (i.e., from 16 h cultures) were spotted onto LB-MOPS-X agar (0.6% agar) plates and incubated anaerobically at 37°C for 24 h. The diameter of the growth halo was used as a measure of swarming. Scanning electron microscopy (SEM) was used to examine the morphology of the extracellular surfaces, while transmission electron microscopy (TEM) and negative staining were used to visualize the flagella of the anaerobically grown WT and *arcA *mutant as previously described [20].

### Pathogenicity studies

For single infectionassays, six to eight week old female C57BL/6 mice bred in the University of Colorado School of Medicine animal facility according to Institutional Animal Care and Use Committee guidelines were used in this study. WT and *arcA *mutant *Salmonella *were grown in LB-MOPS-X broth to stationary phase for about 20 h. For intraperitoneal (i.p.) challenge, two groups of five mice per strain (WT and *arcA *mutant) were inoculated with 250 CFU in 500 μl PBS/mouse. Mortality was scored over a 15- to 30-day period. Competitive infection assays were carried-out as described [33] with modifications. The strains were separately grown overnight in LB broth at 37°C with shaking at 200 rpm. Tetracycline (10 μg/ml) was used to propagate and isolate the *arcA *mutant. Bacterial (i. e.: WT and *arcA *mutant) cultures were diluted in phosphate-buffered saline (PBS) and mixed to produce a 1:1 inoculum ratio. Groups of mice were infected either i.p. or orally (p.o.). Prior to oral infection, food and water were withheld from the mice for 4 h and the bacterial cocktail was administered to the mice by allowing them to drink 20 μl from the end of a pipette tip.

On day 4 or day 6 after i.p. or p.o. infection, respectively, mice were euthanized and mesenteric lymph nodes (MLN), liver and spleen collected for bacterial enumeration. The tissues were homogenized in sterile PBS and 10-fold serial dilutions were plated on LB agar medium with or without 10 μg/mL tetracycline to distinguish the WT (Tet^s^) from the *arcA *mutant (Tet^r^). The number of CFUs of *S*. Typhimurium 14028 s per organ was calculated by subtracting the number of CFU/ml on the LB-Tet plates from the number of CFU/ml on the corresponding LB plates. The competitive index (CI) was calculated as the ratio of the CFU of *arcA *mutant to the CFU of the WT strain recovered from the spleen, liver, and mesenteric lymph nodes (i.e.; CI = [*arcA *mutant/WT]_output_/[*arcA *mutant/WT]_input_).

## Results

### Bacterial growth kinetics

The growth kinetics of the WT and the *arcA *mutant strains were determined under anaerobic conditions in LB-MOPS-X. The *arcA *mutant strain grew at a slower rate than the WT strain. The doubling-times of the WT and *arcA *mutant were 37.0 ± 0.4 and 55.4 ± 0.1 min under anaerobic conditions. Due to the difference in the doubling-times of the two strains, cells used for RNA isolation and subsequent transcriptome profiling were allowed to grow for an equal number of generations (~five generations: OD_600 _= 0.30-0.35) instead of an equal length of time.

### Anaerobic transcriptome profiling

Out of 4,579 genes, the two-tailed Student's *t *test, produced a set of 2,026 coding sequences showing a significant difference between the *arcA *mutant and the WT (p < 0.05). We restricted the analyses to only include highly affected genes (i.e., has a ratio ≥ 2.5-fold) as previously described [20]. Under this constraint, 392 genes were differentially expressed in the *arcA *mutant relative to the WT and, therefore, regulated directly or indirectly by ArcA. Of these, 147 genes were up-regulated and 245 genes were down-regulated (Additional file 1: Table S1). All the genes showing significant differential expression were classified into clusters of orthologous groups (COGs) [34-36] as defined by the National Center for Biotechnology Information (NCBI) http://www.ncbi.nlm.nih.gov/COG (Table 3). It should be noted that throughout the study we compared the levels of transcription in the *arcA *mutant to that in the WT strain. Thus, genes repressed by ArcA posses positive values (i.e., >1), while genes activated by ArcA have negative values (i.e., <1).

**Table 3:** Classification of ArcA regulated genes according to Clusters of Orthologous Groups (COGs)

**Functional Gene Groups**^**a**^	**# of Genes**^**b**^	
	
	ArcA-activated	ArcA-repressed
Cell division and chromosome partitioning	0	0

Cell envelope and biogenesis, outer membrane	4	4

Cell motility and secretion	1	12

Posttranslational modification, protein turnover, chaperones	1	3

Inorganic ion transport and metabolism	1	12

Signal transduction mechanisms	5	3

**Cellular processes^c^**	**12**	**34**

**Defense Mechanisms^c^**	**1**	**1**

Translation, ribosomal structure, and biogenesis	0	7

Transcription	8	18

DNA replication, recombination, and repair	2	4

**Information storage and processing^c^**	**10**	**29**

**Intracell trafficking^c^**	**0**	**1**

Energy production and conversion	9	18

Amino acid transport and metabolism	25	30

Nucleotide transport and metabolism	7	2

Carbohydrate transport and metabolism	20	16

Coenzyme metabolism	0	2

Lipid metabolism	1	7

Secondary metabolites biosynthesis, transport, and catabolism	12	4

**Metabolism^c^**	**74**	**79**

General function prediction only	8	21

Function unknown	8	24

Poorly characterized	23	67

**Unknown^c^**	**39**	**112**

**Total**	**147**	**245**

^a^The differentially expressed genes were classified according to clusters of orthologous groups (COGs) as defined at http://www.ncbi.nlm.nih.gov/COG.

^b^Number of genes activated or repressed (by having a ratio ≥ ± 2.5-fold) by ArcA.

^c^Bolded functional gene catagories contain a summary of the unbolded COG functional gene groups that are located in each of the previous lines.

### Microarray validation

**Figure 1:**
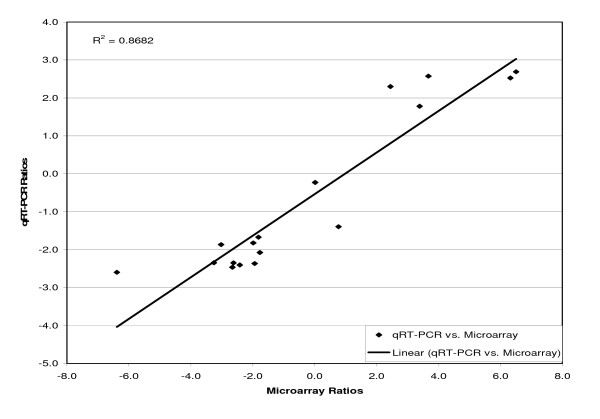
**Correlation between the microarray and the qRT-PCR data of 17 randomly selected genes**. The ratios of changes in gene expression, from the microarray (each *S*. Typhimurium ORF was spotted in triplicate on the slide) and qRT-PCR experiments, for the *arcA *mutant relative to the WT were log_2 _transformed and linearly correlated. The genes selected and the primers used in qRT-PCR are listed in Table 2. Three amplifications of each of the 17 genes were made using 1:5:25 dilutions of the total RNA.

Normalized mRNA levels from qRT-PCR are shown in Table 2. The microarray and qRT-PCR data were log_2 _transformed and plotted (Figure 1). The correlation between the two sets of data was 0.87 (p < 0.05).

### Logo graph and promoter analysis

**Figure 2:**
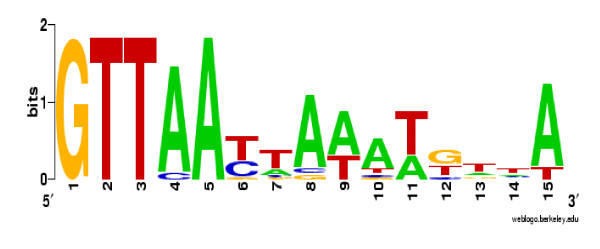
**Logo of the information matrix obtained from the alignment of ArcA sequences for *S*. Typhimurium**. Sequences were obtained by searching the *S*. Typhimurium LT2 genome [Accession #: AE006468 (chromosome) and AE606471 (plasmid)] with known ArcA sequences derived from the corresponding ArcA-regulated genes in *E. coli*. A total of 20 *E*. *coli *sequences were used to obtain the logo shown. The total height of each column of characters represents the amount of information [measured in bits, which is the maximum entropy for the given sequence type (ex. Log_2 _4 = 2 bits for DNA/RNA and log_2 _20 = 4.3 bits for proteins)] for that specific position and the height of each individual character represents the frequency of each nucleotide.

To determine whether a binding site for ArcA might be present in the region upstream of the candidate ArcA-regulated genes, we searched the 5' regions of these highly affected genes (i.e., has a ratio ≥ ± 2.5-fold) for the presence of a putative ArcA-binding motif using a *Salmonella *logo graph (Figure 2) and found 155 genes contained potential ArcA binding sites. Furthermore, fifty-five out of the 147 ArcA-activated genes (37%), and 100 out of the 245 ArcA-repressed genes (41%) contained at least one putative ArcA-binding site (Additional file 1: Table S1).

### ArcA as a repressor

Transcription of the genes required for aerobic metabolism, energy generation, amino acid transport, and fatty acid transport were anaerobically repressed by ArcA (Additional file 1: Table S1). In particular, the genes required for cytochrome-o-oxidase, succinyl-CoA synthetase, glutamate/aspartate transport, trehalose-6-phosphate biosynthesis, long-chain fatty acids transport, spermidine/putrescine transport, dipeptide transport, the genes encoding the two-component tricarboxylic transport system and the site-specific DNA factor for inversion stimulation (*fis*) were among the highest repressed by ArcA. Genes required for L-lactate transport and metabolism, phosphate transport, acetyl-CoA transferase, APC family/D-alanine/D-serine/glycine transport, putative cationic amino acid transporter, peptide methionine sulfoxide reductase, multiple antibiotic resistance operon, as well as many poorly characterized genes were also repressed by ArcA (Additional file 1: Table S1).

**Figure 3:**
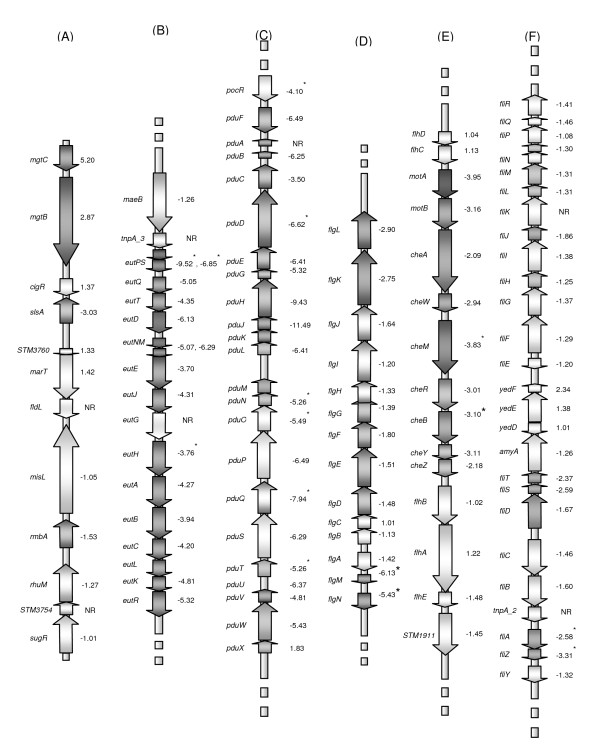
**Organization of major genes for (A) SPI-3, (B) ethanolamine utilization, (C) propanediol utilization, and (D-F) flagellar biosynthesis and motility**. The names of genes are listed to the left of the arrow, an (*) next to the gene indicates the presence of at least one ArcA motif in the 5' - region, the numbers listed to the right of the arrows indicates the ratio of gene expression in the *arcA *mutant relative to that in the WT, and the shading corresponds to the level of gene expression. NR = no expression ratio.

Additionally, some genes related to *Salmonella *virulence were repressed by ArcA. For example, the expression of the *mgtCB *operon (member of SPI-3) that is required for Mg^2+ ^transport/growth in low-magnesium and involved in systemic infections in mice/intramacrophage survival [37-40], genes constituting the lambdoid prophage Gifsy-1 that contributes to the virulence of *S*. Typhimurium [41], and genes coding for a leucine-rich repeat protein (*sspH2*) that is translocated by and coordinately regulated with the SPI-2 TTSS [42] were highly repressed by ArcA (Figure 3A and Additional file 1: Table S1).

### ArcA as an activator

Several of the genes involved in regulating flagellar biosynthesis, motility, chemotaxis, sugar transport, metabolism, and glycogen biosynthesis were found to be anaerobically activated by ArcA (Figure 3D-F and Additional file 1: Table S1). In particular, several of the middle (class 2) flagellar genes and late flagellar (class 3) genes had lower transcript levels in the *arcA *mutant than in the WT strain (Figure 3D-F). There was no significant difference in the transcript levels of the early flagellar genes (class 1) *flhD *and *flhC*, whose gene products FlhD/FlhC are the master regulators of flagellar biosynthesis (Figure 3E). Additionally, several newly identified flagellar genes [43] (i. e., *mcpA*, *mcpC*, and *cheV*) had lower expression levels in the *arcA *mutant than in the WT (Additional file 1: Table S1), while the expression of *mcpB *was not affected. Furthermore, genes coding for transcriptional repressor CytR, nitrite reductase, 2-dexoyribose-5-phosphate aldolase, thymidine phosphorylase, lysine/cadaverine transport protein, putrescine/ornithine antiporter, ornithine decarboxylase, ethanolamine operon, and propanediol operon as well as its transcriptional regulator PocR were activated by ArcA (Figure 3B and 3C, and Additional file 1: Table S1).

The expression of SPI-1 associated genes was not affected by a mutation in *arcA*. However, two SPI-3 genes, *slsA*, encoding a putative inner membrane protein required for colonization of chickens and calves [1,44], and STM3784, a putative sugar phosphotransferase, were activated by ArcA as their expression levels were significantly lower in the mutant than in the WT (Figure 3A and Additional file 1: Table S1).

### Phenotype of the *arcA *mutant

Next, we correlated some of the microarray findings with the corresponding phenotypes of the WT and the *arcA *mutant strains.

#### a. Flagellar biosynthesis and swarming motility

**Figure 4:**
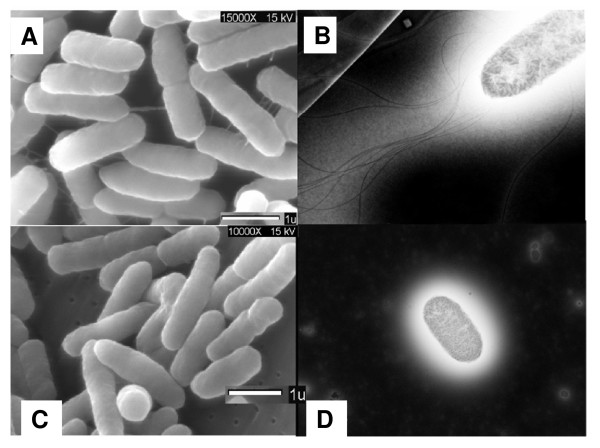
**Comparison of the WT and the *arcA *mutant for surface appendages and flagella via microscopy**. Scanning electron microscopy (SEM) was used to evaluate the WT **(A) **and the *arcA *mutant **(C) **for the presence/absence of surface appendages and negative staining followed by transmission electron microscopy (TEM) was used to evaluate the WT **(B) **and the *arcA *mutant **(D) **for the presence/absence of flagella. Cells were grown anaerobically in LB-MOPS-X media and the samples were prepared as described in Materials and Methods.

The microarray data showed that, in anaerobiosis, the expression of the flagellar biosynthesis, motility, and chemotaxis genes was lower in the *arcA *mutant than in the WT. Therefore, we compared the swarming motility of the WT and the *arcA *mutant in soft agar under anaerobic conditions (Table 4). The data indicated that the *arcA *mutant was ~100% non-motile compared to the WT and that the inclusion of p*arcA *complemented (~57%) this phenotype. We also compared the WT and the *arcA *mutant under anaerobic conditions for the presence of flagella by using SEM (Figure 4A and 4C, left panel) and TEM (Figure 4B and 4D, right panel). The data (Table 4 and Figure 4) clearly showed that the *arcA *mutant Lacked flagella and was non-motile.

**Table 4:** Effect of the *arcA *mutation on swarming motility under anaerobic conditions

	Diameter (cm)	
**Genotype**	**Anaerobic^a^**	**%^b^**

WT	8.0 ± 0.1	100

*arcA *mutant	0.0 ± 0.0	0

Mutant/p*arcA*	4.6 ± 0.1	57

^a ^Average of three replicates.

^b ^Percent relative to the wild-type (WT).

#### b. Virulence in mice

**Figure 5:**
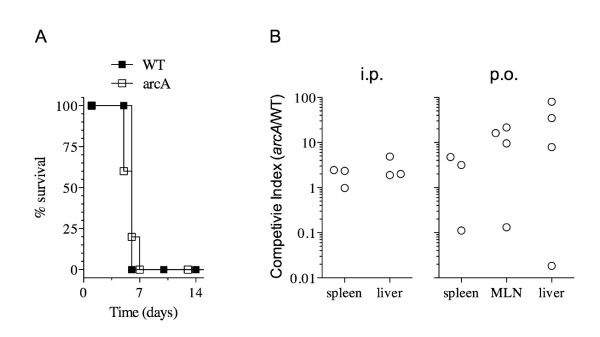
**Virulence comparison of the WT and the *arcA *mutant in 6-8 week old C57BL/6 mice**. **(A) **Single infection assays, where two groups of five mice per strain (WT and *arcA *mutant) were challenged intraperitoneally using 250 CFU/mouse, as described in Materials and Methods. Percent survival is the number of mice surviving relative to the number of mice challenged at zero time; **(B) **Competitive infection assays, where groups of three 6-week-old mice were infected orally (p. o.) or i. p. with a 1:1 mixture of *S*. Typhimurium 14028 s and its isogenic *arcA *mutant. After 4 or 6 days following i.p. or p.o. infection, respectively, mice were euthanized and mesenteric lymph nodes (MLN), liver, and spleen were collected for enumeration of the WT and the mutant. The competitive index (CI) was calculated as described in the Materials and Methods.

The microarray data (Additional file 1: Table S1) showed that ArcA does not significantly regulate the transcription of the virulence genes found in SPI-1, which are important for the ability of *Salmonella *to invade host epithelial cells [2,3,45-47]. However, few virulence genes related to SPI-2 (*sspH2*) and SPI-3 (*mgtCB*, *slsA*, STM3784) were affected by ArcA. Therefore, to evaluate these findings, we tested the virulence of the *arcA *mutant in a murine model of mucosal and acute infection using immunocompetent C57BL/6 mice. The *arcA *mutant was as virulent as the WT strain when 250 CFU/mouse were inoculated via i.p. (Figure 5A). Since intramacrophage survival and replication of *Salmonella *permits the colonization of the spleen and liver of mice [4,48], a further virulence comparison of the WT and the *arcA *mutant was performed using a mixed infection assay. The data showed that the *arcA *mutant had a moderate competitive survival advantage in the reticuloendothelial system compared to the WT in all systemic organs examined following a p.o. or i.p. mixed infection (Figure 5B). In the majority of the mice, the *arcA *mutant was isolated in higher numbers than the WT, although these increases were not statistically significant (p > 0.05). The data generated with the competitive assays is in agreement with i.p. infection data, where the mice succumbed with similar kinetics after infection with *arcA *or WT bacteria.

## Discussion

Although there are several reports on the regulation of specific genes by ArcA in non-virulent strains of *E*. *coli *[6-9,13-17,49-56] and in *Salmonella *spp. [57]. This is the first genome-wide study on the regulatory role of ArcA in *S*. Typhimurium (14028s) under anaerobic conditions. ArcA was found to directly or indirectly control the expression of at least 392 genes. In particular, we showed that ArcA is involved in energy metabolism, flagella biosynthesis, and motility. Additionally, the *arcA *mutant was as virulent as the WT, although it was non-motile. Furthermore, prior to the present report, none of the virulence genes (i. e., SPI-3 and Gifsy-1) had been identified as part of the *Salmonella *ArcA regulon. Finally, several genes involved in metabolism previously identified as being regulated by ArcA in *E*. *coli *[5-17,49-52] were also identified in the present study (Additional file 1: Table S1).

### Logo comparison

In a recent study, a logo was used to graphically compare multiple ArcA sequence alignments of *Shewanella oneidensis *[58] to that of *E*. *coli *[12]. The analysis revealed subtle changes in base pairs at each position between the sequences. Although the ArcA binding motifs of *S. oneidensis *and *E. coli *were similar, the *arcA *regulons and the physiological function of ArcA in these two organisms were different [58]. When comparing the ArcA logos of *E*. *coli *and *S*. *oneidensis *to the one generated herein for *S*. Typhimurium, we found that there is similarity between *S*. Typhimurium and both *E*. *coli *and *S*. *oneidensis*. However, while there is very little variation between the nucleotide sequences at each base pair of *S*. Typhimurium and *E*. *coli*, there is much more variation between *S*. Typhimurium and *S*. *oneidensis*. Therefore, when comparing the genes regulated by ArcA in these three organisms, it is evident that the ArcA regulons of *E*. *coli *and *S*. Typhimurium are more similar than that of *S*. *oneidensis*.

### ArcA and carbon metabolism

Comparing our microarray data in *S*. Typhimurium to the published data of *E*. *coli *[5,12], there are several aspects pertaining to metabolic regulation that are similar between these two organisms. Anaerobically, several ArcA-repressed genes identified in our microarray data are involved in metabolism and transport, while ArcA-activated genes included those coding for enzymes involved in glycogen synthesis and catabolism as well as those for gluconeogenesis. Expression of many of these genes was consistent with those reported in *E*. *coli *[5,9,11-14,52], *H*. *influenzae *[59], and *S*. *oneidensis *[60]. The genes of the two-component tricarboxylic transport system (*tctE*, STM2786, STM2787, STM2788, and STM2789) were the most highly repressed by ArcA (Additional file 1: Table S1). This was not surprising since transport systems for substrates of aerobic pathways have been suggested to be candidates for regulation by ArcA [14]. A similar pattern of anaerobic regulation of these enzymes has also been seen in our previous global analysis of Fnr [20] (Additional file 1: Table S2).

In *E*. *coli*, the CsrA (carbon storage regulator) protein acts post-transcriptionally to balance carbon flow in the cell by activating genes involved in glycolysis and repressing genes participating in gluconeogenesis [61]. In the present study, we found that the transcription of *csrA *was not affected by a mutation in *arcA*, presumably CsrA remained fully functional in the mutant to provide the switch from glycolysis to gluconeogenesis by repressing the genes associated with glycolysis and activating those genes affiliated with gluconeogenesis. A mutation in *arcA *caused a 2.65-fold increase in the expression of *ptsG*, a glucose-specific IIB component of the PTS-system (STM1203), which is required for the first step in glucose metabolism. A similar 2-fold increase was noticed in *E. coli *and the binding of ArcA to the promoter of *ptsG *was demonstrated [54]. Under anaerobic conditions and in the absence of electron acceptors, where the reduced quinone carriers can activate ArcA, it seems to be more advantageous for *S*. Typhimurium and *E*. *coli *cells to control the rate of glucose metabolism in order to reduce the rate of production of acidic end-products. Thus, the adaptation to anaerobic environments requires the regulation of the rate of glycolysis, the utilization of the fermentation products, and the use of the tricarboxylic acid cycle and the glyoxylate shunt in order for the organism to compete with others during sudden changes in oxygen concentrations.

*E*. *coli *contains two oxidases in its respiratory chain. The first, which is known to decrease under anaerobic growth conditions and has a low affinity for oxygen, cytochrome o (encoded by the *cyoABCDE*) and the second, which is known to increase during anaerobic growth and has a high affinity for oxygen, cytochrome *d *(encoded by the *cydAB*) [62]. Our data show that, anaerobically, ArcA repressed the *cyo *operon (Additional file 1: Table S1), while the expression of *cyd *operon was slightly reduced in the *arcA *mutant relative to WT (i.e., ArcA is required for the activation of *cyd*). These results are in agreement with previous reports showing that a mutation in either *arcA *or *arcB *diminished *cyd *operon expression under aerobic and anaerobic conditions, while either mutation did not fully abolish repression of the *cyo *operon anaerobically [55].

Our data showed that the *arcA *mutant has a longer doubling time compared to the WT under anaerobiosis. This result is supported by our microarray data whereby several genes responsible for glycogen synthesis and catabolism as well as those for gluconeogenesis were down-regulated in the *arcA *mutant compared to the WT, while those genes regulating the tricarboxylic acid cycle (TCA), glyoxylate shunt, glycolysis, pentose phosphate shunt, and acetate metabolism were all up-regulated in the *arcA *mutant compared to the WT. Thus, under anaerobiosis, in *arcA *mutant cells, an energy imbalance is created, whereby the cells direct products through various metabolic pathways typically used during aerobiosis due to the de-repression of the TCA cycle, which subsequently yields a pool of reducing equivalents that can only be reduced in the presence of electron acceptors. Normally, during anaerobiosis, less energy in the form of ATP is generated. Thus, the *arcA *mutant cells appear to waste a vast amount of energy to express and maintain metabolic pathways that are not required under anaerobiosis, which may contribute to the slower growth rate of the culture. However, further work is required to determine NAD/NADH pools in the *arcA *mutant compared to the WT.

### ArcA and hydrogenases

Hydrogen gas (H_2_) is an important energy source for the survival of pathogens *in vivo *[63] and is produced in the host via colonic bacterial fermentations [64]. Our results indicated that the *hyb *operon was activated in the *arcA *mutant, but these levels were not within our ± 2.5-fold threshold. Additionally, STM1538, STM1539, STM1786, STM1788, STM1790, and STM1791, which also code for hydrogenases were significantly repressed in the *arcA *mutant (Additional file 1: Table S1), in agreement with previous results [65].

### ArcA regulation of cobalamine synthesis and metabolism

Propanediol (encoded by the *pdu *operon), a fermentation product of rhamnose or fucose [66,67], and ethanolamine (encoded by the *eut *operon), an essential component of bacterial and eukaryotic cells, can be used by *Salmonella *as carbon and energy sources in the mammalian gastrointestinal tract [67]. Vitamin B_12_, its synthesis being encoded by the *cob *operon, is required for the metabolism of ethanolamine and propanediol, while anaerobic utilization of these substrates also requires the use of tetrathionate (*ttr*) as a terminal electron acceptor [68]. The positive regulatory protein, PocR, is necessary for the induction of the *cob *and *pdu *operons and is subject to global regulatory control via ArcA and/or Crp [69,70].

*In vivo *expression technology (IVET) has shown that genes coding for cobalamine synthesis and 1,2-propanediol degradation are required for *Salmonella *replication in macrophages [71], that *pdu *genes may be necessary for intracellular proliferation within the host [72], and that *pdu *mutations, but not *cob *mutations can be attributed to a defect in virulence [73,74]. Strains harboring mutations in ethanolamine utilization genes are attenuated in macrophages and in BALB/c mice when delivered orally, but not intraperitoneally [75].

Our data (Additional file 1: Table S1) show that *pocR*, the transcriptional regulator of propanediol utilization, was significantly activated by ArcA. Furthermore, all of the genes in the *eut *and *pdu *operons were activated by ArcA (Figure 3 and Additional file 1: Table S1). An *arcA *mutation in *S*. Typhimurium has been shown to cause reduced expression of the *cob *and *pdu *operons during anaerobic growth [69]. Interestingly, when comparing the data from the present study and our Fnr data [20], we found that ArcA and Fnr share in the regulation of the *eut *operon (Additional file 1: Table S2), while the *pdu *operon was more positively regulated by ArcA than by Fnr. Transcription of tetrathionate (*ttr *operon) was activated at equal levels by both Fnr and ArcA. Previous studies [68,70] have shown that induction of the *ttr *operon is affected by Fnr, but not by ArcA. This may suggest that Fnr plays a more significant role in regulating the *eut *operon [70], while ArcA acts more significantly on regulating the genes associated with the *pdu *operon. Although, both the *cob *and *pdu *operons were both activated in the *arcA *mutant, this may be due to the effects of *arcA *on anaerobic *pocR *expression, which subsequently regulates the rest of each of these operons.

### ArcA and flagellar biosynthesis/swarming motility/chemotaxis

Our data show that, anaerobically, ArcA positively regulates the expression of genes involved in flagellar biosynthesis, swarming motility, and chemotaxis (Figures 3 and 4; Table 3 and Additional file 1: Table S1) including many newly identified flagellar genes (i.e., *mcpAC *and *cheV*) [43]. Previously, we found that Fnr positively regulates many of the same the flagellar and chemotaxis genes under anaerobic conditions [20]; indeed the anaerobic motility phenotype of the *arcA *mutant was indistinguishable from that previously seen with the *fnr *mutant [20]. Furthermore, the expression of the flagellar biosynthesis, motility, and chemotaxis genes under anaerobiosis was more highly activated by Fnr than by ArcA (Additional file 1: Table S2).

A plethora of regulators affect the expression of *flhDC *and motility in *E*. *coli *and *S*. Typhimurium [20,76-86]. Our data showed that ArcA activates class 2 and class 3 flagellar genes and we identified a potential ArcA binding site in *filA*, *filZ*, *flgM*, and *flgN*. ArcA seems to slightly repress *flhDC *(i. e., below our cut-off level of ±2.5-fold). In agreement with our work, ArcA was recently shown to be necessary for the expression of *fliA *in *E*. *coli*, but not for the master regulator, *flhDC *[56]. However, using *in silico *analysis, the authors did not identify ArcA binding sites in the promoter regions of *fliA *or other class 2 flagellar genes [56],

### ArcA and antioxidant defenses

Under aerobic conditions, ArcA has been reported to be essential for the resistance of *S*. Enteritidis to RNS and ROS via an unknown mechanism [57]. In agreement with this report [57], we found that the *arcA *mutant of *S*. Typhimurium to be more sensitive to hydrogen peroxide (H_2_O_2_) under aerobic conditions (Additional file 1: Figure S2). Anaerobically, our data indicate that the expression of many of the antioxidant genes [i.e.: *sodA*, *sodB*, *sodC1*, and *sodC2 *(coding for superoxide dismutases) and *katG *and *katE *(coding for hydroperoxidases), and *hmpA *(coding for flavohemoglobin)] were not significantly affected by ArcA; however the expression of STM1731 (Mn-catalase, *katN*) was significantly increased in the *arcA *mutant compared to the WT (Additional file 1: Table S1). To date, the physiological role of Mn-catalase (KatN) in *S*. Typhimurium has not been thoroughly examined, although, its ability to scavenge H_2_O_2 _and work together with KatE, KatG, and the hydroperoxide reductases to protect the cell from oxidative stress has been demonstrated [87]. In addition, it has been proposed that the substitution of iron by manganese as a co-factor might be a way to circumvent iron restriction by the host during infection [88].

### ArcA and pathogenesis

The majority of the virulence factors (~200 genes) of *S*. Typhimurium are chromosomally located within *Salmonella *pathogenicity islands (SPIs) [2,89-93]. SPI-1 and SPI-2 both encode TTSSs [4,45,94]. SPI-1 effectors' proteins are required for epithelial cell invasion [95], while SPI-2 encodes secreted proteins, their specific chaperones [4], and a two-component regulatory system [96,97], are all required for intracellular replication. Recently, SPI-1 invasion genes were found to be required for intramacrophage survival [98] and systemic infection in mice [99]. Our data have shown that most of the SPI-1 through SPI-5 genes were not significantly regulated by ArcA, with the exception of three genes contained within SPI-3 including, *mgtC*, *mgtB*, and *slsA *(Figure 3 and Additional file 1: Table S1). Thus, it is not surprising that our *arcA *mutant was determined to be as virulent as the WT strain following individual infection studies (Figure 5A), but was slightly more persistent than the WT following o. p. and i. p. competitive infection studies (Figure 5B), however, the difference was not statistically significant (p > 0.05).

Flagellar regulons have been shown to influence virulence gene expression in several pathogenic microorganisms [100-106]. Interestingly, data from our previous study [20], showed that the *fnr *mutant was non-motile and non-virulent, while in the present study, the *arcA *mutant was non-motile, but remained virulent. Clearly, the lack of motility does not necessarily correlate with the lack of virulence in *S*. Typhimurium.

### Overlapping global regulation by ArcA and Fnr

ArcA and Fnr are two well known redox regulators in *E*. *coli*, *S*. Typhimurium, and other bacteria. We previously published the first report on the global role of Fnr in anaerobically grown *S*. Typhmurium [20]. The present study is the first report on the global regulatory role of ArcA in the same organism under the same experimental conditions and statistical constraints. Therefore, it is possible and reliable to compare genes/operons regulated by these two important transcriptional factors (i. e., ArcA and Fnr). The data indicated that ArcA and Fnr shared in the regulation of 120 genes; while the numbers of genes solely regulated by either ArcA or Fnr were 272 and 191, respectively. The 120 genes that were regulated by either ArcA or Fnr are listed (Additional file 1: Table S2). These include genes involved in cytochrome c oxidase (*cyoABCDE*), glutamate/aspartate transport (*gltlJKL*), dipeptide transport (*dppABCDF*), succinyl-CoA synthetase - α & β subunits (*sucDC*), tricarboxylic transport (STM2786, STM2787, STM2788), L-lactate transport and metabolism (*lldPRD*), nitrite reductase (*nrfAB*), 2-dexoyribose-5-phosphate aldolase (*deoC*), thymidine phosphorylase (*deoA*), aerotaxis sensor receptor (*aer*), ethanolamine utilization (*eut *operon - STM2454-2470), and several genes involving flagellar biosynthesis, motility, and chemotaxis. Interestingly, most of the 120 genes were regulated by ArcA and Fnr in the same fashion (i.e., repressed or activated) except for *yneB *(putative fructose-1,6-bisphosphate aldolase - STM4078), which was activated by ArcA, but repressed by Fnr (Additional file 1: Table S2). The opposing regulation of *yneB *by ArcA and Fnr indeed warrant further studies.

## Conclusion(s)

Herein, we report on the role of the two-component regulator, ArcA, in the genome-wide response to oxygen in *Salmonella*. Our data clearly demonstrate that ArcA serves, directly or indirectly, as a regulator/modulator of genes involved in aerobic/anaerobic energy metabolism and motility. In a recent study [20], we demonstrated that the oxygen sensing, global regulator, Fnr participates in coordinating anaerobic metabolism, flagellar biosynthesis, motility, chemotaxis, and virulence in *S*. Typhimurium. In the present study, we identified a set of 120 genes whose regulation is shared between ArcA and Fnr. We also demonstrated that Fnr plays a more hierarchical role than ArcA in pathogenesis. Furthermore, under our experimental conditions, we demonstrated that the lack of motility does not necessarily correspond to the lack of virulence in *S*. Typhimurium.

## Authors' contributions

MRE transduced and confirmed the *arcA *mutant, constructed and confirmed the complement plasmid (p*arcA*), performed the Western blotting, performed the growth studies in aerobic and anaerobic conditions, compared our microarray data to that in other studies, conducted the qRT-PCR and lethality studies in the presence of hydrogen peroxide, participated in the motility studies, and contributed to the writing/editing of the manuscript. RCF conducted the microarrays and performed the analysis, constructed the logo, participated in motility studies, and contributed to the editing of the manuscript. AV-T and JJ-C carried-out all of the mice studies. MM and SP constructed and provided the microarray slides. HMH conceived the idea, directed the research, and contributed to the writing/editing of the manuscript. All authors have read and approved the final manuscript.

## Supplementary Material

Additional file 1 **Analysis of the ArcA regulon in anaerobically grown *Salmonella enterica sv*. Typhimurium**. Identification of ArcA by Western blot; Effects of H_2_O_2 _on viability of the ArcA mutant; List of genes differentially regulated by ArcA; and List of genes shared with the Fnr regulon. A. Supplemental Methods: Western blot analysis of ArcA. H_2_O_2 _survival assays. B. Supplemental Figures: Figure S1. Western blot of total proteins of the WT, *arcA *mutant, and *arcA*^-^/p*arcA *complement strains. Figure S2. Effects of hydrogen peroxide on viability of the WT and the *arcA *mutant under anerobiosis. C. Supplemental Tables: Table S1. Differentially expressed genes and the presence/absence of putative ArcA-binding motifs in their 5' regions. Table S2. Comparison of the 120 genes shared between the ArcA and the Fnr regulons of *S*. Typhimurium under anaerobiosis. Click here for file
