# Supplementary material for: Analysis of the ArcA regulon in anaerobically grown Salmonella enterica sv. Typhimurium
Source: BMC Microbiol. 2011 Mar 21;11:58. doi: 10.1186/1471-2180-11-58 (PMC3075218; doi:10.1186/1471-2180-11-58)
Supplement: Additional file 1 — Analysis of the ArcA regulon in anaerobically grown Salmonella enterica sv. Typhimurium. Identification of ArcA by Western blot; Effects of H2O2 on viability of the ArcA mutant; List of genes differentially regulated by ArcA; and List of genes shared with the Fnr regulon. A. Supplemental Methods: Western blot analysis of ArcA. H2O2 survival assays. B. Supplemental Figures: Figure S1. Western blot of total proteins of the WT, arcA mutant, and arcA-/parcA complement strains. Figure S2. Effects of hydrogen peroxide on viability of the WT and the arcA mutant under anerobiosis. C. Supplemental Tables: Table S1. Differentially expressed genes and the presence/absence of putative ArcA-binding motifs in their 5' regions. Table S2. Comparison of the 120 genes shared between the ArcA and the Fnr regulons of S. Typhimurium under anaerobiosis. [file 1471-2180-11-58-S1.DOC]

**ADDITIONAL FILES**

**Additional File 1**

**Title: Analysis of the ArcA regulon in anaerobically grown *Salmonella enterica sv*. Typhimurium**

**Description: Identification of ArcA by Western blot; Effects of H_2_O_2_ on viability of the ArcA mutant; List of genes differentially regulated by ArcA; and List of genes shared with the Fnr regulon**

1. **Supplemental Methods**

**Western blot analysis of ArcA**

Cells [WT, *arcA* mutant (NC980), and *arcA* complemented with p*arcA* (NC989)] were collected, by centrifugation (13,000 X g for 3 min.), from aerobically grown cultures (5 ml at OD_600_ = 0.6); and pellets were resuspended in 2X Lammeli buffer (BioRad, Hercules, CA). The suspension was heated in a boiling water bath for 5 min. The same amount of protein from each extract (WT, *arcA* mutant, and *arcA* complemented with p*arcA*) were separated on a 10% SDS-PAGE gel using a Bio-Rad Mini-Protean II electrophoresis system (Bio-Rad) and electroblotted to a 0.20 µm PVDF membrane (Invitrogen, Carlsbad, CA) using the Invitrogen XCell II blot module (Invitrogen). Transfer efficiency of proteins were verified via Coomassie blue staining (Bio-Rad) of the SDS-PAGE gel according to the manufacturer’s instructions. The membrane was blocked for 3 hr at room temperature (RT) with 3% bovine serum albumin (BSA) (Sigma) solubilized in TBST [1X Tris-phosphate buffered saline (pH = 8.0), 0.05% Tween 20]. Primary goat polyclonal antibodies specific for the *E*. *coli* ArcA (SfrA) protein (A generous gift from Dr. Philip Silverman, Department of Botany and Microbiology, University of Oklahoma) [49] were added to fresh blocking buffer at a 1:10,000 dilution and incubated with gentle agitation for 2 hrs at room temperature (RT). The membrane was subsequently washed three times each for 30 min at RT in TBST, after which fresh blocking buffer containing 1:5,000 alkaline-phosphatase-conjugated protein G was added to the membrane and incubated for 3 hr at RT. After final incubation, the membrane was washed twice in TBST (15 min. each). The blot was developed with the pre-mixed NBT-BCIP (nitroblue tetrazolium-5-bromo-4-chloro-3-indolylphosphate toluidinium) (Sigma) alkaline phosphatase substrate. Conjugated protein G and substrate were obtained from the Pierce Chemical Co., Rockford, IL. The protein content of every sample was confirmed by running a protein gel in parallel with the one that was used for Western blotting and subsequently staining that gel with Coomassie blue. The transfer efficiency of the protein gel used for the Western blot was confirmed by staining for the proteins in the remaining gel with Coomassie blue.

**H_2_O_2_ survival assays**

The survival of the WT strain and its isogenic *arcA* mutant were compared in the presence of hydrogen peroxide (H_2_O_2_). Each culture (50 ml) was grown aerobically, with shaking at 250 rpm, in LB-MOPS-X broth at 37°C to an OD_600_ = 0.6. Under these conditions, the doubling-times of the WT and the mutant were 22.6 ± 0.1 and 33.0 ± 0.2 min, respectively. The cultures were subsequently treated with chloramphenicol (60 µg/ml) and incubated at 37°C with shaking at 250 rpm for 15 min, to stop further growth and protein biosynthesis during H_2_O_2_ challenge. Cells were then centrifuged at 5,000 X g for 10 min at 4˚C, washed and re-suspended in 5 ml of fresh LB-MOPS-X with chloramphenicol (60 µg/ml). Equal concentrations of each strain were used to inoculate two culture flasks each containing LB-MOPS-X with chloramphenicol (60 µg/ml) and 0 mM and 8.25 mM H_2_O_2_ (Fisher Scientific, Fair Lawn, NJ). The flasks were incubated at 37˚C for 60 min with shaking at 250 rpm. At 0, 15, 30, 45, and 60 min of exposure in presence/absence of H_2_O_2_, aliquots of each strain were serially diluted in LB-MOPS-X containing excess catalase (Sigma) to remove the residual H_2_O_2_, spread onto LB-MOPS-X agar plates, and incubated aerobically for 24 hr at 37˚C prior to enumerating the colonies. Each point represents the mean of three independent values each from four separate experiments.

1. **Supplemental Figures**

1 2 3 4

kDa

198

98

62

49

38

28

17

14

6

3


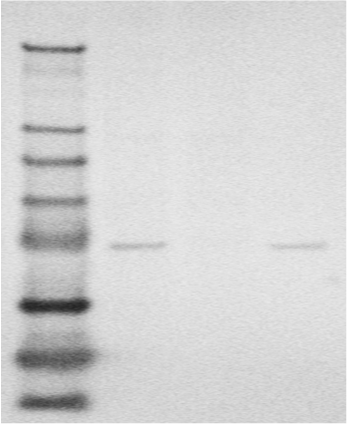


ArcA

**Figure S1: Western blot of total proteins of the WT, *arcA* mutant, and *arcA*^-^/p*arcA* complement strains.** Separation of standardized total proteins was performed on a 10% denaturing SDS-PAGE gel. Following electroblotting, *S*. Typhimurium ArcA was probed for with *E*. *coli* ArcA polyclonal antibodies. Lane 1, protein molecular weight marker (3-198 kDa); lane 2, WT *S*. Typhimurium 14028s; lane 3, *arcA* mutant (NC 980); lane 4, *arcA*^-^/p*arcA* complement strain (NC 989). The protein content of every sample was confirmed by running a protein gel in parallel with the one that was used for Western blotting and subsequently staining that gel with Coomassie blue. The transfer efficiency of the protein gel used for the Western blot was confirmed by staining the proteins in the remaining gel with Coomassie blue.

**A**

**B**

**Figure S2:** **Effects of hydrogen peroxide on viability of the WT and the *arcA* mutant under aerobiosis.** Cells were grown aerobically to exponential phase, treated with chloramphenicol (60 µg/ml) to stop growth and halt protein biosynthesis, and washed in LB-MOPS-X with chloramphenicol. Cells were subsequently exposed to 8.25 mM of hydrogen peroxide (H_2_O_2_) for 60 min with aliquots taken every 15 min, serially diluted in LB-MOPS-X containing catalase, and plated onto LB-MOPS-X. The cells were grown in LB-MOPS-X broth and exposed to H_2_O_2_ in LB-MOPS-X broth containing chloramphenicol (60 µg/ml). WT, □; *arcA* mutant, ■.

1. **Supplemental Tables**

**Table S1. Differentially expressed genes and the presence/absence of putative ArcA-binding motifs in their 5’ regions**

| **Locus^a^** | **Category^b^** | **Name^c^** | **STM Gene Function^d^** | **t value^e^** | **DF^f^** | **Prob t^g^** | **Ratio^h^** | **Strand^i^** | **Start^j^** | **End^k^** | **Sequence^l^** | **Score^m^ Score^m^** | **In (P)^n^** |
| --- | --- | --- | --- | --- | --- | --- | --- | --- | --- | --- | --- | --- | --- |
| **PSLT019** |  | pefB | plasmid-encoded fimbriae; regulation | -7.700 | 10.00 | 2.00E-05 | -2.74 | R | -27 | -41 | ttttGTACATGAAAAGACAtata | 4.35 | -7.12 |
| **PSLT042** |  | PSLT042 | putative integrase protein; K07494 putative transposase | 12.670 | 6.00 | 2.00E-05 | 3.62 |  |  |  |  |  |  |
| **PSLT047** |  | PSLT047 | putative cytoplasmic protein | 10.550 | 5.00 | 8.00E-05 | 5.07 | D | -256 | -270 | tggtGTTAATGCTAATGTAcgca | 7.49 | -9.51 |
| **PSLT048** |  | tlpA | alpha-helical coiled-coil protein | 8.903 | 6.00 | 1.00E-04 | 2.78 | R | -73 | -87 | tggtGTTAATGCTAATGTAcgca | 7.49 | -9.51 |
| **PSLT049** |  | PSLT049 | putative DNA polymerase III epsilon subunit (3'-5' exonuclease) | 8.612 | 6.00 | 2.00E-04 | 2.67 |  |  |  |  |  |  |
| **PSLT050** |  | PSLT050 | hypothetical protein | 11.630 | 9.00 | 1.00E-06 | 2.59 |  |  |  |  |  |  |
| **PSLT076** |  | traY | DNA-binding protein | -4.050 | 5.00 | 9.00E-03 | -2.62 | D | -292 | -306 | ttgtGTTAAGTGAATGTTAatta | 9.87 | -11.91 |
| **PSLT088** |  | traC | ATP-binding protein | 3.950 | 7.00 | 6.00E-03 | 2.98 |  |  |  |  |  |  |
| **PSLT106** |  | PSLT106 | homologue of mvpA, Shigella flexneri | 10.920 | 6.00 | 3.00E-05 | 2.65 |  |  |  |  |  |  |
| **R460032** |  |  |  | -3.540 | 4.00 | 2.20E-02 | -4.50 |  |  |  |  |  |  |
| **STM0001** | E | thrL | thr operon leader peptide; K08278 thr operon leader peptide | -9.620 | 9.00 | 3.00E-06 | -3.45 | R | -211 | -225 | ctaaGTCAATAAATTTTTAaatt | 6.38 | -8.6 |
| **STM0002** | E | thrA | aspartokinase I , bifunctional enxyme N-terminal is aspartokinaseI and C-terminal is homoserine dehydrogenase I | -18.500 | 10.00 | 7.00E-09 | -3.79 | R | -64 | -78 | ctaaGTCAATAAATTTTTAaatt | 6.38 | -8.6 |
| **STM0018** | G | STM0018 | putative exochitinase | 4.248 | 6.00 | 6.00E-03 | 5.04 | D | -71 | -85 | gataTTTAATTACATGATAaatg | 6.8 | -8.94 |
| **STM0039** | P | nhaA | NhaA familiy of transport protein, Na+/H antiporter, pH dependent; K03313 Na+:H+ antiporter, NhaA family | 31.350 | 10.00 | 3.00E-11 | 4.01 |  |  |  |  |  |  |
| **STM0040** | K | nhaR | transcriptional activator of nhaA (LysR family); K03717 LysR family transcriptional regulator, transcriptional activator of nhaA | 9.851 | 6.00 | 1.00E-04 | 2.62 |  |  |  |  |  |  |
| **STM0068** | - | caiF | transcriptional regulator of cai and fix operon; K08277 transcriptional activator CaiF | -24.000 | 9.00 | 2.00E-09 | -2.85 |  |  |  |  |  |  |
| **STM0096** | K | hepA | RNA polymerase associated protein, putative SNF2 family RNA helicase [EC:3.6.1.-]; K03580 ATP-dependent helicase HepA | 8.530 | 6.00 | 2.00E-04 | 2.90 | R | -308 | -322 | cagcGTTGACCAAGTGTAAaagg | 5.45 | -7.89 |
| **STM0169** | G | gcd | glucose dehydrogenase [EC:1.1.5.2]; K00117 quinoprotein glucose dehydrogenase | 9.930 | 7.00 | 3.00E-05 | 3.05 | D | -243 | -257 | acatGTTAATTCCTATACTtaat | 4.73 | -7.38 |
| **STM0175** | N | stiC | putativie fimbrial usher | 4.877 | 10.00 | 7.00E-04 | 2.71 |  |  |  |  |  |  |
| **STM0256** | K | yafC | putative transcriptional regulator | 4.564 | 6.00 | 4.00E-03 | 2.60 |  |  |  |  |  |  |
| **STM0289** | S | STM0289 | putative cytoplasmic protein | 2.437 | 7.00 | 4.50E-02 | 4.47 |  |  |  |  |  |  |
| **STM0309** | I | yafH | putative acyl-CoA dehydrogenase [EC:1.3.99.-]; K06445 acyl-CoA dehydrogenase | 8.622 | 5.00 | 3.00E-04 | 6.19 | D | -213 | -227 | ttttGTAAACACATTGCTAaaaa | 4.21 | -7.02 |
| **STM0314** |  | STM0314 | pseudogene; frameshift relative to *Escherichia* *coli* probable peptide chain release factor (GB:AAC73340.1) | 11.630 | 10.00 | 4.00E-07 | 2.97 |  |  |  |  |  |  |
| **STM0315** | J | prfH | putative peptide chain release factor; K02839 peptide chain release factor RF-H | 5.763 | 7.00 | 8.00E-04 | 4.00 |  |  |  |  |  |  |
| **STM0356** | G | STM0356 | putative inner membrane protein; K08178 MFS transporter, SHS family, lactate transporter | 11.220 | 6.00 | 2.00E-05 | 4.71 | D | -127 | -141 | ctttGTTAATAATATGTAAtgat | 11.2 | -13.81 |
| **STM0398** | T | phoR | sensory kinase in two-component regulatory system with PhoB, regulates pho regulon [EC:2.7.13.3]; K07636 two-component system, OmpR family, phosphate regulon sensor histidine kinase PhoR | 7.498 | 7.00 | 2.00E-04 | 4.03 |  |  |  |  |  |  |
| **STM0439** | H | cyoE | protohaeme IX farnesyltransferase (haeme O biosynthesis) [EC:2.5.1.-]; K02301 protoheme IX farnesyltransferase | 7.042 | 6.00 | 5.00E-04 | 4.79 |  |  |  |  |  |  |
| **STM0440** | C | cyoD | cytochrome o ubiquinol oxidase subunit IV; K02300 cytochrome o ubiquinol oxidase operon protein cyoD | 14.490 | 6.00 | 7.00E-06 | 5.76 | R | -50 | -64 | taccGTTAACAATCAGGTGatgg | 4.54 | -7.24 |
| **STM0441** | C | cyoC | cytochrome o ubiquinol oxidase subunit III [EC:1.10.3.-]; K02299 cytochrome o ubiquinol oxidase subunit III | 12.690 | 6.00 | 1.00E-05 | 5.47 |  |  |  |  |  |  |
| **STM0442** | C | cyoB | cytochrome o ubiquinol oxidase subunit I [EC:1.10.3.-]; K02298 cytochrome o ubiquinol oxidase subunit I | 3.676 | 5.00 | 1.30E-02 | 3.58 | R | -156 | -170 | atttGTTAATAACGTCCTTgaac | 4.31 | -7.09 |
| **STM0443** | C | cyoA | cytochrome o ubiquinol oxidase subunit II [EC:1.10.3.-]; K02297 cytochrome o ubiquinol oxidase subunit II | 4.233 | 5.00 | 7.00E-03 | 3.68 | D | -50 | -64 | aattGTTAAGTAATTGTTTtatt | 8.9 | -10.81 |
| **STM0467** |  | ffs | signal recognition particle, RNA component; K01983 component of ribonucleoprotein particle | -4.770 | 6.00 | 3.00E-03 | -3.35 |  |  |  |  |  |  |
| **STM0581** | K | STM0581 | putative regulatory protein | 8.655 | 6.00 | 1.00E-04 | 5.65 |  |  |  |  |  |  |
| **STM0582** | - | ybdJ | putative inner membrane protein | 4.630 | 6.00 | 3.00E-03 | 3.59 | R | -280 | -294 | ctttGTTAAACGTAAGTTAaaac | 5.21 | -7.71 |
| **STM0600** | T | cstA | carbon starvation protein; K06200 carbon starvation protein | 3.613 | 5.00 | 1.50E-02 | 2.66 | D | -230 | -244 | tgttGTTAATTACGGTAAAggtg | 6.38 | -8.6 |
| **STM0634** | K | ybeF | putative transcriptional regulator | 4.939 | 7.00 | 1.00E-03 | 2.80 |  |  |  |  |  |  |
| **STM0650** | G | STM0650 | putative hydrolase C-terminus [EC:4.2.1.7]; K01685 altronate hydrolase | -14.300 | 5.00 | 2.00E-05 | -8.13 |  |  |  |  |  |  |
| **STM0662** | E | gltL | ABC superfamily (atp_bind), glutamate/aspartate transporter [EC:3.6.3.-]; K10004 glutamate/aspartate transport system ATP-binding protein | 20.990 | 5.00 | 4.00E-06 | 12.11 |  |  |  |  |  |  |
| **STM0663** | E | gltK | ABC superfamily (membrane), glutamate/aspartate transporter; K10002 glutamate/aspartate transport system permease protein | 23.800 | 6.00 | 8.00E-07 | 7.23 | R | -80 | -94 | ccagGTTCATCATCTCTGAcgtc | 5.88 | -8.21 |
| **STM0664** | E | gltJ | ABC superfamily (membrane), glutamate/aspartate transporter; K10003 glutamate/aspartate transport system permease protein | 26.970 | 5.00 | 8.00E-07 | 8.47 |  |  |  |  |  |  |
| **STM0665** | E | gltI | ABC superfamily (bind_prot), glutamate/aspartate transporter; K10001 glutamate/aspartate transport system substrate-binding protein | 4.031 | 5.00 | 1.00E-02 | 4.52 | D | -218 | -232 | aaatGTTAACAAACACACAtaac | 7.05 | -9.14 |
| **STM0699** | R | STM0699 | putative cytoplasmic protein | -6.180 | 5.00 | 2.00E-03 | -4.88 |  |  |  |  |  |  |
| **STM0700** | E | potE | APC family, putrescine/ornithine antiporter; K03756 putrescine:ornithine antiporter | -4.710 | 5.00 | 5.00E-03 | -3.94 |  |  |  |  |  |  |
| **STM0701** | E | speF | ornithine decarboxylase isozyme [EC:4.1.1.17]; K01581 ornithine decarboxylase | -4.700 | 5.00 | 5.00E-03 | -3.95 |  |  |  |  |  |  |
| **STM0729** | R | abrB | putative transport protein | 5.904 | 5.00 | 1.00E-03 | 2.56 |  |  |  |  |  |  |
| **STM0738** | C | sucC | succinyl-CoA synthetase, beta subunit [EC:6.2.1.5]; K01903 succinyl-CoA synthetase beta chain | 3.304 | 5.00 | 2.00E-02 | 2.84 |  |  |  |  |  |  |
| **STM0739** | C | sucD | succinyl-CoA synthetase, alpha subunit [EC:6.2.1.5]; K01902 succinyl-CoA synthetase alpha chain | 4.378 | 5.00 | 7.00E-03 | 3.65 | R | -304 | -318 | ctttATTAATTAAAACGGAcatt | 5.51 | -7.93 |
| **STM0790** |  | hutU | pseudogene; frameshift relative to Pseudomonas putida urocanate hydratase; HUTU (SW:P25080) | 4.524 | 5.00 | 6.00E-03 | 5.72 |  |  |  |  |  |  |
| **STM0791** | E | hutH | histidine ammonia lyase [EC:4.3.1.3]; K01745 histidine ammonia-lyase | 11.020 | 5.00 | 9.00E-05 | 6.57 | D | -330 | -344 | ggcaGTTAAGTCTCTCTCAactg | 6.06 | -8.35 |
| **STM0793** | H | bioA | 7,8-diaminopelargonic acid synthetase [EC:2.6.1.62]; K00833 adenosylmethionine-8-amino-7-oxononanoate aminotransferase | 4.169 | 8.00 | 3.00E-03 | 2.59 | R | -277 | -291 | acttGTAAACCAAATTAAAaaga | 5.55 | -7.97 |
| **STM0813** | R | ybhP | putative cytoplasmic protein | 8.455 | 9.00 | 2.00E-05 | 3.04 |  |  |  |  |  |  |
| **STM0837** | S | ybiS | putative periplasmic protein | 11.830 | 9.00 | 8.00E-07 | 2.79 |  |  |  |  |  |  |
| **STM0838** | R | ybiT | putative ABC transporter ATPase component | 9.439 | 5.00 | 1.00E-04 | 2.82 |  |  |  |  |  |  |
| **STM0841** | - | ybiU | putative cytoplasmic protein | 18.210 | 7.00 | 3.00E-07 | 3.04 | R | -220 | -234 | taagGTTAATGCAAGTTTAaaat | 7.89 | -9.87 |
| **STM0842** | R | ybiV(1) | putative hydrolase of the HAD superfamily [EC:3.1.3.23]; K07757 sugar-phosphatase | 7.853 | 6.00 | 3.00E-04 | 3.20 | D | -106 | -120 | taagGTTAATGCAAGTTTAaaat | 7.89 | -9.87 |
| **STM0858** | C | STM0858 | putative dehydrogenase [EC:1.5.5.1]; K00311 electron-transferring-flavoprotein dehydrogenase | 3.549 | 7.00 | 9.00E-03 | 3.04 | D | 25 | 11 | cgcgGGTAACTATAATAGAatgc | 5.91 | -8.23 |
| **STM0877** | E | potF | ABC superfamily (peri_perm), putrescine transporter; K02055 spermidine/putrescine transport system substrate-binding protein | 7.479 | 9.00 | 3.00E-05 | 2.73 | R | -7 | -21 | aaagGTTAATAGCAGCATAaaaa | 5.12 | -7.65 |
| **STM0878** | E | potG | ABC superfamily (atp_bind), putrescine transporter [EC:3.6.3.31]; K02052 spermidine/putrescine transport system ATP-binding protein | 9.123 | 7.00 | 4.00E-05 | 4.98 | R | -208 | -222 | taccGTTAATAAATTACTTaccg | 4.16 | -6.99 |
| **STM0907** | R | aSTM0907 | Fels-1 prophage; putative chitinase; K03791 putative chitinase | -6.570 | 9.00 | 8.00E-05 | -2.79 |  |  |  |  |  |  |
| **STM0914** | - | STM0914 | putative phage tail component | 8.090 | 6.00 | 1.00E-04 | 5.36 |  |  |  |  |  |  |
| **STM0915** | - | STM0915 | hypothetical protein | 5.616 | 5.00 | 2.00E-03 | 4.36 |  |  |  |  |  |  |
| **STM0916** | - | STM0916 | putative major tail protein | 12.750 | 5.00 | 4.00E-05 | 18.36 |  |  |  |  |  |  |
| **STM0917** | - | STM0917 | putative minor tail protein | 7.770 | 6.00 | 4.00E-04 | 4.23 |  |  |  |  |  |  |
| **STM0925** | - | STM0925 | putative host-specificity protein | 18.500 | 7.00 | 7.00E-07 | 12.19 |  |  |  |  |  |  |
| **STM0927** | - | STM0927 | Fels-1 prophage; putative tail assembly protein | 11.710 | 5.00 | 8.00E-05 | 47.13 | D | -260 | -274 | cacgGTTAAAAACATTGCAttta | 4.66 | -7.33 |
| **STM0935** | E | poxB | pyruvate dehydrogenase [EC:1.2.2.2]; K00156 pyruvate dehydrogenase (cytochrome) | 13.410 | 8.00 | 8.00E-07 | 2.90 |  |  |  |  |  |  |
| **STM0950** | Q | STM0950 | SlsA | -9.020 | 7.00 | 5.00E-05 | -2.75 | R | -172 | -186 | atcgGTTCATTCAGTGGCTcgcc | 5.51 | -7.93 |
| **STM0952** | K | STM0952 | putative transcriptional regulator | 9.371 | 10.00 | 3.00E-06 | 2.59 | R | -111 | -125 | gttcGTTCACTCTTTTCTTatca | 4.72 | -7.37 |
| **STM0980** | F | cmk | cytidine monophosphate (CMP) kinase [EC:2.7.4.14]; K00945 cytidylate kinase | 7.953 | 8.00 | 4.00E-05 | 2.52 |  |  |  |  |  |  |
| **STM1029** | - | STM1029 | hypothetical protein | 5.354 | 8.00 | 8.00E-04 | 2.62 |  |  |  |  |  |  |
| **STM1034** | - | STM1034 | putative RecA/RadA recombinase | 6.861 | 6.00 | 3.00E-04 | 2.72 |  |  |  |  |  |  |
| **STM1041** | S | STM1041 | probable minor tail protein | 4.872 | 5.00 | 5.00E-03 | 16.54 |  |  |  |  |  |  |
| **STM1046** | R | aSTM1046 | probable tail assembly protein | 2.496 | 9.00 | 3.50E-02 | 2.54 | R | -278 | -292 | acctGTTAAGACAGTTTGTtgat | 4.2 | -7.02 |
| **STM1050** | - | STM1050 | tail fiber assembly like-protein | 8.066 | 5.00 | 5.00E-04 | 65.10 | D | -260 | -274 | cacgGTTAAAAACATTGCAttta | 4.66 | -7.33 |
| **STM1052** |  | STM1052 | pseudogene; in-frame stop following codon 112 | 10.830 | 5.00 | 1.00E-04 | 16.23 |  |  |  |  |  |  |
| **STM1060** | R | STM1060 | putative iron-sulfur protein | 6.426 | 6.00 | 6.00E-04 | 2.75 |  |  |  |  |  |  |
| **STM1101** | Q | hpaG | putative bifunctional enzyme 2-hydroxyhepta-2,4-diene-1,7-dioatesomerase / 5-carboxymethyl-2-oxo-hex-3-ene-1,7-dioatedecarboxylase protein [EC:4.1.1.68 5.3.3.-]; K05921 5-oxopent-3-ene-1,2,5-tricarboxylate decarboxylase / 2-hydroxyhepta-2,4-di • • • | 5.399 | 7.00 | 1.00E-03 | 3.37 | D | -183 | -197 | tattGTTAATCACATCACAaata | 7.28 | -9.34 |
| **STM1122** | K | ycdC | putative transcriptional repressor; K09017 TetR/AcrR family transcriptional regulator | 7.062 | 6.00 | 4.00E-04 | 2.74 |  |  |  |  |  |  |
| **STM1123** | S | STM1123 | putative periplasmic protein | 10.060 | 7.00 | 1.00E-05 | 2.91 | D | -250 | -264 | tgtgGTTAATTAAGGCTCCgtca | 4.15 | -6.98 |
| **STM1124** |  | putA | bifunctional in plasma membrane proline dehydrogenase and pyrroline-5-carboxylate dehydrogenase OR in cytoplasm a transcriptional repressor [EC:1.5.1.12 1.5.99.8]; K00294 1-pyrroline-5-carboxylate dehydrogenase; K00318 proline dehydrogenase | 4.010 | 5.00 | 1.00E-02 | 4.49 |  |  |  |  |  |  |
| **STM1125** | E | putP | SSS family, major sodium/proline symporter; K03307 solute:Na+ symporter, SSS family | 21.910 | 5.00 | 3.00E-06 | 8.50 | D | -67 | -81 | ggtaGTTAACACTTTTAAAaggt | 7.08 | -9.16 |
| **STM1129** | G | STM1129 | putative N-acylglucosamine-6-phosphate 2-epimerase [EC:5.1.3.9]; K01788 N-acylglucosamine-6-phosphate 2-epimerase | -6.360 | 6.00 | 9.00E-04 | -3.77 |  |  |  |  |  |  |
| **STM1130** | S | STM1130 | putative inner membrane protein | -6.120 | 5.00 | 1.00E-03 | -3.50 | D | -186 | -200 | acatGATAACTCCATGTAAttat | 5.84 | -8.19 |
| **STM1131** | - | STM1131 | putative outer membrane protein | -8.620 | 10.00 | 7.00E-06 | -3.15 |  |  |  |  |  |  |
| **STM1132** | G | STM1132 | putative sialic acid transporter | -10.100 | 7.00 | 3.00E-05 | -3.21 |  |  |  |  |  |  |
| **STM1133** | R | STM1133 | putative dehydrogenase | -19.200 | 10.00 | 3.00E-09 | -2.59 |  |  |  |  |  |  |
| **STM1138** | - | ycdZ | putative inner membrane protein | -15.500 | 5.00 | 2.00E-05 | -13.16 | R | -256 | -270 | tgcaGTTAAGGGTGATTTAcatc | 4.42 | -7.16 |
| **STM1139** | M | csgG | putative transcriptional regulator in curly assembly/transport, 2nd curli operon; K06214 curli production assembly/transport component CsgG | -16.100 | 9.00 | 5.00E-08 | -4.22 | D | -106 | -120 | gcttGTTGACCAATATTAAtacc | 4.65 | -7.32 |
| **STM1171** | N | flgN | flagellar biosynthesis: belived to be export chaperone for FlgK and FlgL; K02399 flagella synthesis protein FlgN | -25.600 | 6.00 | 3.00E-07 | -5.43 | R | -313 | -327 | tctgGTCAAGTATTTCTGAcaaa | 4.05 | -6.92 |
| **STM1172** | K | flgM | anegative regulator of flagellin synthesis (anti-sigma factor); K02398 negative regulator of flagellin synthesis FlgM | -11.900 | 5.00 | 6.00E-05 | -6.13 | R | -189 | -203 | atagGTTAATAAGAATATTccca | 5.05 | -7.6 |
| **STM1183** | N | flgK | flagellar biosynthesis, hook-filament junction protein 1; K02396 flagellar hook-associated protein 1 FlgK | -7.380 | 7.00 | 2.00E-04 | -2.75 |  |  |  |  |  |  |
| **STM1184** | N | flgL | flagellar biosynthesis; hook-filament junction protein; K02397 flagellar hook-associated protein 3 FlgL | -10.100 | 5.00 | 1.00E-04 | -2.90 |  |  |  |  |  |  |
| **STM1203** | G | ptsG | Sugar Specific PTS family, glucose-specific IIBCcomponent [EC:2.7.1.69]; K02778 PTS system, glucose-specific IIB component; K02779 PTS system, glucose-specific IIC component | 5.111 | 7.00 | 1.00E-03 | 2.65 |  |  |  |  |  |  |
| **STM1214** | - | ycfR | putative outer membrane protein | 6.504 | 6.00 | 7.00E-04 | 7.20 | D | -253 | -267 | gatcGTTAAGTTAATGCTTacga | 4.8 | -7.42 |
| **STM1225** | E | potB | ABC superfamily (membrane), spermidine/putrescine transporter; K02054 spermidine/putrescine transport system permease protein | 6.305 | 9.00 | 2.00E-04 | 3.06 |  |  |  |  |  |  |
| **STM1226** | E | potA | ABC superfamily (atp_bind), spermidine/putrescine transporter [EC:3.6.3.31]; K02052 spermidine/putrescine transport system ATP-binding protein | 7.805 | 6.00 | 3.00E-04 | 3.23 | R | -248 | -262 | tgtgGTTAACCACCTTAATcact | 4.34 | -7.11 |
| **STM1246** | M | pagC | putative outer membrane protein; K07804 putatice virulence related protein PagC | 18.780 | 5.00 | 4.00E-06 | 4.10 | D | -113 | -127 | accgGTTACCTAAATGAGCgata | 4.08 | -6.94 |
| **STM1279** | K | yeaM | putative regulatory protein | 5.438 | 7.00 | 1.00E-03 | 2.57 |  |  |  |  |  |  |
| **STM1300** | - | STM1300 | putative periplasmic protein | -6.170 | 5.00 | 2.00E-03 | -5.05 |  |  |  |  |  |  |
| **STM1301** | L | STM1301 | putative mutator MutT protein [EC:3.6.1.-]; K08320 CTP pyrophosphohydrolase | -6.490 | 8.00 | 2.00E-04 | -3.08 |  |  |  |  |  |  |
| **STM1303** | E | astC | succinylornithine transaminase, also has acetylornitine transaminase activity [EC:2.6.1.81]; K00840 succinylornithine aminotransferase | 10.050 | 5.00 | 1.00E-04 | 15.60 | R | -261 | -275 | taaaGTTATTTATATGTTAatta | 8.94 | -10.86 |
| **STM1304** | E | astA | arginine succinyltransferase [EC:2.3.1.109]; K00673 arginine N-succinyltransferase | 13.790 | 6.00 | 1.00E-05 | 7.09 |  |  |  |  |  |  |
| **STM1305** | C | astD | succinylglutamic semialdehyde dehydrogenase [EC:1.2.1.71]; K06447 succinylglutamic semialdehyde dehydrogenase | 6.366 | 7.00 | 4.00E-04 | 5.32 |  |  |  |  |  |  |
| **STM1306** | E | astB | succinylarginine dihydrolase [EC:3.5.3.23]; K01484 succinylarginine dihydrolase | 8.493 | 5.00 | 3.00E-04 | 2.88 | D | -104 | -118 | gatcGTTAACTGGAATAAAccgc | 5.31 | -7.79 |
| **STM1324** | S | STM1324 | putative cytoplasmic protein | -11.100 | 10.00 | 7.00E-07 | -2.82 |  |  |  |  |  |  |
| **STM1328** | S | STM1328 | putative outer membrane protein; K09953 hypothetical protein | 10.950 | 6.00 | 2.00E-05 | 2.59 |  |  |  |  |  |  |
| **STM1367** | - | ydiH | putative cytoplasmic protein | 10.450 | 9.00 | 3.00E-06 | 3.10 |  |  |  |  |  |  |
| **STM1368** | R | STM1368 | putative Na+-dicarboxylate symporter | 7.279 | 9.00 | 4.00E-05 | 2.74 |  |  |  |  |  |  |
| **STM1459** | C | STM1459 | putative oxidoreductase, inner membrane protein; K03617 electron transport complex protein RnfA | 6.699 | 8.00 | 2.00E-04 | 3.31 | R | -226 | -240 | acagGTTAACTGTAGGGTTatta | 6.45 | -8.66 |
| **STM1518** | - | marB | multiple antibiotic resistance protein | 5.373 | 6.00 | 1.00E-03 | 3.04 | D | 11 | -3 | gagcGTTCAGGATATTCCAaatg | 4.93 | -7.51 |
| **STM1519** | - | marA | transcriptional activator | 16.570 | 6.00 | 7.00E-06 | 7.13 |  |  |  |  |  |  |
| **STM1520** | K | marR | transcriptional repressor of marRAB operon, multiple antibiotic resistance protein; K03712 MarR family transcriptional regulator | 18.970 | 6.00 | 1.00E-06 | 6.30 | R | -228 | -242 | ataaGTCAACTAAATGAATtggc | 6.38 | -8.6 |
| **STM1538** | C | STM1538 | hydrogenase large chain [EC:1.12.99.6]; K06281 hydrogenase large subunit | 13.820 | 8.00 | 9.00E-07 | 3.87 |  |  |  |  |  |  |
| **STM1539** | C | STM1539 | hydrogenase small chain [EC:1.12.99.6]; K06282 hydrogenase small subunit | 12.180 | 10.00 | 3.00E-07 | 3.32 | R | -143 | -157 | ataaATTAATTTAATGTTAttgg | 5.39 | -7.84 |
| **STM1548** | - | STM1548 | putative S-adenosylmethionine/tRNA-ribosyltransferase-isomerase | 3.741 | 8.00 | 5.00E-03 | 2.54 |  |  |  |  |  |  |
| **STM1625** | K | ydcI | putative transcriptional regulator | 25.840 | 8.00 | 8.00E-09 | 6.35 | D | -220 | -234 | aaatGTTAATAATATTTTGctat | 7.1 | -9.18 |
| **STM1668** | - | STM1668 | hypothetical protein | 3.852 | 9.00 | 4.00E-03 | 3.40 |  |  |  |  |  |  |
| **STM1712** | C | acnA | aconitate hydratase [EC:4.2.1.3]; K01681 aconitate hydratase 1 | 3.442 | 5.00 | 1.80E-02 | 2.70 | D | -210 | -224 | ggttGTTATCAAAACGTTAcatt | 4.75 | -7.4 |
| **STM1728** | R | yciG | putative cytoplasmic protein | 17.160 | 6.00 | 3.00E-06 | 8.26 | R | -40 | -54 | aaacGTTAATGCATTGTTTgttg | 7.48 | -9.5 |
| **STM1729** | S | yciF | putative cytoplasmic protein | 10.940 | 8.00 | 7.00E-06 | 5.94 |  |  |  |  |  |  |
| **STM1730** | S | yciE | putative cytoplasmic protein | 15.240 | 6.00 | 7.00E-06 | 14.20 | R | -302 | -316 | taatGTTCAGTATAATTCAtttt | 7.64 | -9.64 |
| **STM1731** | P | STM1731 | putative catalase; K07217 Mn-containing catalase | 9.255 | 8.00 | 2.00E-05 | 5.12 |  |  |  |  |  |  |
| **STM1771** | P | chaA | CaCA family, sodium-calcium/proton antiporter; K07300 Ca2+:H+ antiporter | 6.660 | 10.00 | 7.00E-05 | 2.81 | R | -265 | -279 | aggtGTTAATATTTTGGAAagag | 5.43 | -7.87 |
| **STM1786** | C | STM1786 | hydrogenase small chain [EC:1.12.99.6]; K06282 hydrogenase small subunit | 8.131 | 6.00 | 2.00E-04 | 3.47 | R | -107 | -121 | tcggATTAACGATAAGAAAcaat | 4.87 | -7.48 |
| **STM1788** | C | STM1788 | putative Ni/Fe-hydrogenase 1 b-type cytochrome subunit; K03620 Ni/Fe-hydrogenase 1 B-type cytochrome subunit | 12.240 | 7.00 | 4.00E-06 | 3.31 | D | -285 | -299 | aagcGTTAAGGAAAAGAACgatg | 4.51 | -7.22 |
| **STM1790** | - | STM1790 | putative thiol-disulfide isomerase and thioredoxins; K03619 hydrogenase-1 operon protein HyaE | 5.973 | 6.00 | 1.00E-03 | 2.67 |  |  |  |  |  |  |
| **STM1791** | - | STM1791 | putative hydrogenase-1 protein; K03618 hydrogenase-1 operon protein HyaF | 8.041 | 8.00 | 4.00E-05 | 2.62 |  |  |  |  |  |  |
| **STM1795** | E | STM1795 | putative glutamic dehyrogenase-like protein [EC:1.4.1.3]; K00261 glutamate dehydrogenase (NAD(P)+) | 3.863 | 5.00 | 1.10E-02 | 4.04 | R | -180 | -194 | agtgGTTAACTATCCGCTAtaag | 5.99 | -8.29 |
| **STM1802** | M | dadX | alanine racemase 2, catabolic [EC:5.1.1.1]; K01775 alanine racemase | 8.923 | 5.00 | 3.00E-04 | 8.23 |  |  |  |  |  |  |
| **STM1803** | E | dadA | D-amino acid dehydrogenase subunit [EC:1.4.99.1]; K00285 D-amino-acid dehydrogenase | 6.878 | 5.00 | 1.00E-03 | 10.52 | D | -250 | -264 | ctggGTTATCAAGATGTAAtcag | 4.23 | -7.04 |
| **STM1817** | J | rnd | RNase D, processes tRNA precursor [EC:3.1.26.3]; K03684 ribonuclease D | 10.430 | 5.00 | 1.00E-04 | 3.34 |  |  |  |  |  |  |
| **STM1818** | I | fadD | acyl-CoA synthetase (long-chain-fatty-acid--CoA ligase) [EC:6.2.1.3]; K01897 long-chain fatty-acid-CoA ligase | 20.040 | 6.00 | 1.00E-06 | 6.67 | D | -226 | -240 | ctgaGTTAATATAATGTTAacga | 8.58 | -10.5 |
| **STM1834** | S | yebN | putative transport protein | 5.337 | 5.00 | 3.00E-03 | 5.70 |  |  |  |  |  |  |
| **STM1835** | Q | rrmA | 23S rRNA m1G745 methyltransferase [EC:2.1.1.51]; K00563 rRNA (guanine-N1-)-methyltransferase | 6.278 | 10.00 | 1.00E-04 | 3.20 | R | -297 | -311 | acagGTAAACGACATGACAactc | 4.49 | -7.21 |
| **STM1850** | J | yebU | putative rRNA methyltransferase | 9.387 | 6.00 | 1.00E-04 | 2.52 |  |  |  |  |  |  |
| **STM1851** | - | STM1851 | putative cytoplasmic protein | 8.928 | 5.00 | 2.00E-04 | 3.47 |  |  |  |  |  |  |
| **STM1857** | K | STM1857 | putative acetyltransferase | 8.483 | 9.00 | 2.00E-05 | 4.17 | R | -237 | -251 | ataaGTTAATTAAATGTATcaat | 11.15 | -13.72 |
| **STM1867** | - | pagK | PhoPQ-activated gene; K07801 hypothetical protein PagK | 4.758 | 5.00 | 4.00E-03 | 2.64 | D | -178 | -192 | ttaaGTTAAATATTTTATAaatg | 6.8 | -8.94 |
| **STM1868** | R | mig-3 | phage-tail assembly-like protein | 10.800 | 7.00 | 1.00E-05 | 3.38 |  |  |  |  |  |  |
| **STM1880** | S | yebE | putative inner membrane protein | 7.395 | 5.00 | 5.00E-04 | 2.72 |  |  |  |  |  |  |
| **STM1881** | - | yebF | putative periplasmic protein | 19.300 | 6.00 | 8.00E-07 | 4.06 |  |  |  |  |  |  |
| **STM1882** | S | yebG | DNA damage-inducible protein; K09918 hypothetical protein | 10.330 | 6.00 | 3.00E-05 | 3.50 |  |  |  |  |  |  |
| **STM1896** | - | STM1896 | putative cytoplasmic protein | 12.800 | 8.00 | 2.00E-06 | 6.88 |  |  |  |  |  |  |
| **STM1916** | T | cheY | chemotaxis regulator, transmits chemoreceptor signals to flagelllar motor components; K03413 two-component system, chemotaxis family, response regulator CheY | -6.570 | 6.00 | 8.00E-04 | -3.11 |  |  |  |  |  |  |
| **STM1917** | N | cheB | protein-glutamate methylesterase [EC:3.1.1.61]; K03412 protein-glutamate methylesterase, two-component system, chemotaxis family, response regulator CheB | -10.400 | 5.00 | 9.00E-05 | -3.10 | D | -68 | -82 | ctccGTTAATTTATTGGAAaagc | 7.13 | -9.2 |
| **STM1918** | N | cheR | chemotaxis protein methyltransferase [EC:2.1.1.80]; K00575 chemotaxis protein methyltransferase CheR | -13.400 | 7.00 | 5.00E-06 | -3.01 |  |  |  |  |  |  |
| **STM1919** | N | cheM | methyl accepting chemotaxis protein II, aspartate sensor-receptor; K05875 methyl-accepting chemotaxis protein II, aspartate sensor receptor | -8.290 | 6.00 | 3.00E-04 | -3.83 | R | -295 | -309 | tacgGTTAAACATAAGGCAcctt | 5.42 | -7.86 |
| **STM1920** | N | cheW | purine-binding chemotaxis protein; regulation; K03408 purine-binding chemotaxis protein CheW | -7.070 | 6.00 | 5.00E-04 | -2.94 |  |  |  |  |  |  |
| **STM1922** | N | motB | Chemotaxis MotB protein; K02557 chemotaxis MotB protein | -10.400 | 6.00 | 3.00E-05 | -3.16 |  |  |  |  |  |  |
| **STM1923** | N | motA | proton conductor component of motor, torque generator; K02556 chemotaxis MotA protein | -14.900 | 5.00 | 2.00E-05 | -3.95 |  |  |  |  |  |  |
| **STM1927** | T | yecG | putative universal stress protein | -6.260 | 6.00 | 1.00E-03 | -2.59 | R | 3 | -11 | tactGTTTACTAAAGGTAAaata | 7.3 | -9.35 |
| **STM1928** | G | otsA | trehalose-6-phosphate synthase [EC:2.4.1.15]; K00697 alpha,alpha-trehalose-phosphate synthase (UDP-forming) | 7.857 | 6.00 | 2.00E-04 | 3.30 |  |  |  |  |  |  |
| **STM1929** | G | otsB | trehalose-6-phosphate phophatase, biosynthetic [EC:3.1.3.12]; K01087 trehalose-phosphatase | 11.340 | 6.00 | 3.00E-05 | 2.95 |  |  |  |  |  |  |
| **STM1950** | K | sdiA | transcriptional regulator of ftsQAZ gene cluster (LuxR/UhpA family); K07782 LuxR family transcriptional regulator | -7.540 | 5.00 | 6.00E-04 | -3.47 | R | -139 | -153 | cgagGTTAATAATCTGGCAgaat | 8.27 | -10.21 |
| **STM1955** | - | fliZ | putative regulator of FliA; K02425 FliZ protein | -9.300 | 6.00 | 9.00E-05 | -3.31 | D | -110 | -124 | tggtGTTAACGCTGTATTAccag | 4.18 | -7.01 |
| **STM1956** | K | fliA | sigma F (sigma 28) factor of RNA polymerase, transcription of late flagellar genes (class 3a and 3b operons); K02405 RNA polymerase sigma factor for flagellar operon FliA | -4.730 | 6.00 | 4.00E-03 | -2.58 | D | -301 | -315 | tatcGTGAATTCACTGTATaccg | 4.16 | -6.99 |
| **STM1961** | O | fliS | flagellar biosynthesis; repressor of class 3a and 3b operons (RflA activity); K02422 flagellar protein FliS | -7.280 | 6.00 | 3.00E-04 | -2.59 |  |  |  |  |  |  |
| **STM2001** | S | yeeI | putative inner membrane protein; K09933 hypothetical protein | -9.050 | 7.00 | 4.00E-05 | -3.04 |  |  |  |  |  |  |
| **STM2036** | K | pocR | Propanediol utilization: transcriptional regulation, AraC family | -12.200 | 5.00 | 5.00E-05 | -4.10 | R | -272 | -286 | ctcaGTTAATTTATTGTTAtaaa | 8.58 | -10.5 |
| **STM2037** | G | pduF | Propanediol utilization: propanediol diffusion facilitator | -11.100 | 5.00 | 9.00E-05 | -6.49 |  |  |  |  |  |  |
| **STM2039** | E | pudB | Propanediol utilization: polyhedral bodies | -6.110 | 5.00 | 2.00E-03 | -6.25 |  |  |  |  |  |  |
| **STM2040** | Q | pduC | glycerol dehydratase large subunit [EC:4.2.1.30]; K06120 glycerol dehydratase large subunit | -4.470 | 5.00 | 6.00E-03 | -3.50 |  |  |  |  |  |  |
| **STM2041** | - | pduD | Propanediol utilization: dehydratase, medium subunit [EC:4.2.1.30]; K06121 glycerol dehydratase medium subunit | -6.200 | 5.00 | 2.00E-03 | -6.62 | D | -177 | -191 | tgcgGTTAATGACGTCAATgact | 4.05 | -6.92 |
| **STM2042** | Q | pduE | Propanediol utilization: dehydratase, small subunit [EC:4.2.1.30]; K06122 glycerol dehydratase small subunit | -8.110 | 5.00 | 4.00E-04 | -6.41 |  |  |  |  |  |  |
| **STM2043** | - | pduG | propanediol dehydratase reactivation protein | -5.990 | 5.00 | 2.00E-03 | -5.32 |  |  |  |  |  |  |
| **STM2044** | - | pduH | propanediol dehydratase reactivation protein | -10.700 | 5.00 | 1.00E-04 | -9.43 |  |  |  |  |  |  |
| **STM2045** | Q | pduJ | polyhedral body protein | -14.800 | 5.00 | 2.00E-05 | -11.49 |  |  |  |  |  |  |
| **STM2046** | Q | pduK | polyhedral body protein | -16.300 | 5.00 | 1.00E-05 | -10.53 |  |  |  |  |  |  |
| **STM2047** | Q | pduL | propanediol utilization protein | -9.080 | 5.00 | 2.00E-04 | -6.41 |  |  |  |  |  |  |
| **STM2048** | - | pduM | propanediol utilization protein | -5.850 | 5.00 | 2.00E-03 | -4.85 |  |  |  |  |  |  |
| **STM2049** | Q | pduN | polyhedral body protein | -5.770 | 5.00 | 2.00E-03 | -5.26 | R | -274 | -288 | cctgCTTAATTAATTGAATattc | 5.02 | -7.58 |
| **STM2050** | S | pduO | propanediol utilization protein | -10.700 | 5.00 | 1.00E-04 | -5.49 | R | 11 | -3 | cctgCTTAATTAATTGAATattc | 5.02 | -7.58 |
| **STM2051** | C | pduP | CoA-dependent propionaldehyde dehydrogenase | -6.210 | 5.00 | 2.00E-03 | -6.49 |  |  |  |  |  |  |
| **STM2052** | C | pduQ | Propanediol utilization: propanol dehydrogenase [EC:1.1.1.-]; K00100 | -11.800 | 5.00 | 7.00E-05 | -7.94 | R | -276 | -290 | ctcaGTTAGCGAATAGAAAagcc | 4.69 | -7.35 |
| **STM2053** | C | pduS | polyhedral body protein | -6.460 | 5.00 | 1.00E-03 | -6.29 |  |  |  |  |  |  |
| **STM2054** | Q | pduT | polyhedral body protein | -8.930 | 6.00 | 9.00E-05 | -5.26 | D | -294 | -308 | agagGTTAACCATGTCTCAggct | 7.1 | -9.18 |
| **STM2055** | E | pduU | Propanediol utilization: polyhedral bodies | -7.050 | 5.00 | 7.00E-04 | -6.37 |  |  |  |  |  |  |
| **STM2056** | E | pduV | Propanediol utilization | -7.290 | 5.00 | 6.00E-04 | -4.81 |  |  |  |  |  |  |
| **STM2057** | C | pduW | Propanediol utilization: propionate kinase [EC:2.7.2.15]; K00932 propionate kinase | -7.470 | 5.00 | 6.00E-04 | -5.43 |  |  |  |  |  |  |
| **STM2136** | O | yegQ | putative protease [EC:3.4.-.-]; K08303 putative protease | 8.753 | 7.00 | 5.00E-05 | 3.09 |  |  |  |  |  |  |
| **STM2163** | E | yehX | putative ABC-type proline/glycine betaine transport system, ATPase component; K05847 osmoprotectant transport system ATP-binding protein | 9.990 | 10.00 | 2.00E-06 | 2.76 |  |  |  |  |  |  |
| **STM2183** | F | cdd | cytidine deaminase [EC:3.5.4.5]; K01489 cytidine deaminase | -5.620 | 5.00 | 2.00E-03 | -13.16 |  |  |  |  |  |  |
| **STM2186** | E | STM2186 | putative NADPH-dependent glutamate synthase beta chain or related oxidoreductase | -4.710 | 5.00 | 5.00E-03 | -4.00 | R | -136 | -150 | atttGTTCCTTATATCATAataa | 5.57 | -7.98 |
| **STM2187** | F | yeiA | dihydropyrimidine dehydrogenase | -4.630 | 5.00 | 5.00E-03 | -3.92 | D | -303 | -317 | tcatGTTAACAAAAGATTTgtct | 5.01 | -7.57 |
| **STM2207** | G | setB | proton efflux pump; K03291 MFS transporter, SET family, sugar efflux transporter | 8.151 | 5.00 | 3.00E-04 | 8.13 | D | -186 | -200 | ccctGGTAACAAACTGGAAtgct | 4.99 | -7.56 |
| **STM2208** | - | STM2208 | putative inner membrane protein | 17.140 | 8.00 | 1.00E-07 | 5.98 |  |  |  |  |  |  |
| **STM2209** | - | STM2209 | putative inner membrane protein | 4.319 | 7.00 | 3.00E-03 | 3.27 |  |  |  |  |  |  |
| **STM2219** | R | yejF | putative ATPase component of ABC-type transport system, contain duplicated ATPase domain; K02031 peptide/nickel transport system ATP-binding protein; K02032 peptide/nickel transport system ATP-binding protein | 4.499 | 6.00 | 5.00E-03 | 4.13 |  |  |  |  |  |  |
| **STM2220** | - | yejG | putative cytoplasmic protein | 12.940 | 10.00 | 2.00E-07 | 3.44 | D | -248 | -262 | atgtGTTAATATAAATGTAagta | 6.26 | -8.5 |
| **STM2241** | S | sspH2 | leucine-rich repeat protein | 11.670 | 5.00 | 6.00E-05 | 4.99 |  |  |  |  |  |  |
| **STM2243** | - | STM2243 | putative tail fiber protein of phage | 13.580 | 7.00 | 4.00E-06 | 4.55 |  |  |  |  |  |  |
| **STM2244** | - | STM2244 | virulence protein | 12.720 | 10.00 | 2.00E-07 | 3.28 |  |  |  |  |  |  |
| **STM2314** | T | STM2314 | putative chemotaxis signal transduction protein [EC:2.7.3.-]; K03415 two-component system, chemotaxis family, response regulator CheV | -12.100 | 5.00 | 6.00E-05 | -5.75 |  |  |  |  |  |  |
| **STM2315** | R | yfbK | putative von Willebrand factor, vWF type A domain | -6.500 | 7.00 | 3.00E-04 | -2.93 | R | -152 | -166 | aaaaGATAATTATATTTTTgagg | 6.34 | -8.57 |
| **STM2340** | G | STM2340 | putative transketolase [EC:2.2.1.1]; K00615 transketolase | 10.200 | 6.00 | 7.00E-05 | 4.24 | D | -218 | -232 | agcaGTTAACGAATTCTCAccac | 8.27 | -10.2 |
| **STM2341** | G | STM2341 | putative transketolase [EC:2.2.1.1]; K00615 transketolase | 13.120 | 6.00 | 1.00E-05 | 4.28 | R | -292 | -306 | ttacGTTCATTCTTTCGCTccgc | 4.24 | -7.04 |
| **STM2342** | S | STM2342 | PTS system, unknown pentitol phosphotransferase enzyme IIC component; K03475 PTS system, unknown pentitol phosphotransferase enzyme IIC component | 13.890 | 6.00 | 1.00E-05 | 4.45 | R | -70 | -84 | tttcTTTAATACTTTTTTAatgt | 4.11 | -6.96 |
| **STM2343** | G | STM2343 | putative sugar phosphotransferase component IIB [EC:2.7.1.69]; K02822 PTS system, unknown pentitol phosphotransferase enzyme IIB component | 12.450 | 7.00 | 5.00E-06 | 3.48 |  |  |  |  |  |  |
| **STM2344** | G | STM2344 | putative phosphotransferase system [EC:2.7.1.69]; K02821 PTS system, unknown pentitol phosphotransferase enzyme IIA component | 14.500 | 7.00 | 2.00E-06 | 4.27 | D | -267 | -281 | gtttGTTCATAATTTCATAcacc | 7.23 | -9.3 |
| **STM2355** | E | argT | ABC superfamily (bind_prot), lysine/arginine/ornithine transport protein; K10013 lysine/arginine/ornithine transport system substrate-binding protein | 9.254 | 5.00 | 2.00E-04 | 3.52 | R | -168 | -182 | atttGTTAATAAAACGTTGcaat | 5.07 | -7.61 |
| **STM2391** | I | fadL | transport of long-chain fatty acids; sensitivity to phage T2; K06076 long-chain fatty acid transport protein | 26.330 | 5.00 | 6.00E-07 | 6.23 | D | -20 | -34 | agtcGTTAATGGTGGGAAAcgcg | 4.63 | -7.31 |
| **STM2401** | M | ddg | lipid A biosynthesis lauroyl acyltransferase | 4.514 | 7.00 | 3.00E-03 | 2.65 |  |  |  |  |  |  |
| **STM2409** | F | nupC | NUP family, nucleoside transport; K03317 concentrative nucleoside transporter, CNT family | -5.050 | 5.00 | 3.00E-03 | -3.11 | D | -180 | -194 | tacaGATCACTAATTTTGAatct | 5.16 | -7.68 |
| **STM2454** | K | eutR | putative regulator ethanolamine operon (AraC/XylS family); K04033 AraC family transcriptional regulator, ethanolamine operon transcriptional activator | -13.000 | 5.00 | 4.00E-05 | -5.32 |  |  |  |  |  |  |
| **STM2455** | Q | eutK | putative carboxysome structural protein, ethanolamine utilization; K04025 ethanolamine utilization protein EutK | -16.200 | 6.00 | 5.00E-06 | -4.81 |  |  |  |  |  |  |
| **STM2456** | E | eutL | putative carboxysome structural protein, ethanolamine utilization; K04026 ethanolamine utilization protein EutL | -15.300 | 5.00 | 1.00E-05 | -5.13 |  |  |  |  |  |  |
| **STM2457** | E | eutC | ethanolamine ammonia-lyase, light chain [EC:4.3.1.7]; K03736 ethanolamine ammonia-lyase small subunit | -7.610 | 5.00 | 6.00E-04 | -4.20 |  |  |  |  |  |  |
| **STM2458** | E | eutB | ethanolamine ammonia-lyase, heavy chain [EC:4.3.1.7]; K03735 ethanolamine ammonia-lyase large subunit | -6.030 | 5.00 | 2.00E-03 | -3.94 |  |  |  |  |  |  |
| **STM2459** | E | eutA | CPPZ-55 prophage; chaperonin in ethanolamine utilization; K04019 ethanolamine utilization protein EutA | -12.000 | 6.00 | 2.00E-05 | -4.27 |  |  |  |  |  |  |
| **STM2460** | E | eutH | putative transport protein, ethanolamine utilization; K04023 ethanolamine transporter | -10.200 | 6.00 | 8.00E-05 | -3.76 | D | 35 | 21 | gggcGTTAACCAATAAGAAatcg | 4.65 | -7.32 |
| **STM2462** | E | eutJ | putative ethanolamine utilization protein; K04024 ethanolamine utilization protein EutJ | -12.600 | 7.00 | 6.00E-06 | -4.31 |  |  |  |  |  |  |
| **STM2463** | C | eutE | putative aldehyde oxidoreductase in ethanolamine utilization; K04021 aldehyde dehydrogenase | -4.940 | 5.00 | 4.00E-03 | -3.70 |  |  |  |  |  |  |
| **STM2464** | Q | eutN | putative detox protein in ethanolamine utilization; K04028 ethanolamine utilization protein EutN | -11.000 | 5.00 | 7.00E-05 | -6.29 |  |  |  |  |  |  |
| **STM2465** | Q | eutM | putative detox protein in ethanolamine utilization; K04027 ethanolamine utilization protein EutM | -6.860 | 5.00 | 1.00E-03 | -5.07 |  |  |  |  |  |  |
| **STM2466** | C | eutD | putative phosphotransacetylase in ethanolamine utilization; K04020 ethanolamine utilization protein EutD | -11.000 | 6.00 | 6.00E-05 | -6.13 |  |  |  |  |  |  |
| **STM2467** | E | eutT | putative cobalamin adenosyltransferase, ethanolamine utilization [EC:2.5.1.17]; K04032 ethanolamine utilization cobalamin adenosyltransferase | -5.560 | 5.00 | 3.00E-03 | -4.35 |  |  |  |  |  |  |
| **STM2468** | E | eutQ | putative ethanolamine utilization protein; K04030 ethanolamine utilization protein EutQ | -5.130 | 5.00 | 4.00E-03 | -5.05 |  |  |  |  |  |  |
| **STM2469** | E | eutP | putative ethanolamine utilization protein; K04029 ethanolamine utilization protein EutP | -7.430 | 5.00 | 7.00E-04 | -6.85 | D | -273 | -287 | gtgaGTTAACAAAAAGTTAattg | 11.56 | -14.46 |
| **STM2470** | E | eutS | putative carboxysome structural protein, ethanol utilization; K04031 ethanolamine utilization protein EutS | -9.080 | 5.00 | 3.00E-04 | -9.52 | R | -193 | -207 | ggtaGATCACTAAGAGAAAgaaa | 4.44 | -7.18 |
| **STM2511** | R | guaB | inositol-5-monophosphate dehydrogenase [EC:1.1.1.205]; K00088 IMP dehydrogenase | 5.461 | 7.00 | 1.00E-03 | 2.72 | R | -265 | -279 | ggagGTTAATAAATATTGCcgcg | 5.17 | -7.68 |
| **STM2526** | F | ndk | nucleoside diphosphate kinase [EC:2.7.4.6]; K00940 nucleoside-diphosphate kinase | 8.373 | 8.00 | 2.00E-05 | 2.66 | D | -206 | -220 | ttatTTTAAAAAAATGTTAcctg | 4.53 | -7.24 |
| **STM2558** | E | cadB | APC family, lysine/cadaverine transport protein; K03757 cadaverine:lysine antiporter | -11.400 | 6.00 | 4.00E-05 | -3.29 | D | -132 | -146 | atatGTTAATTCAAAAAAAtcaa | 6.66 | -8.82 |
| **STM2585** | - | STM2585 | transposase-like protein | 15.710 | 7.00 | 2.00E-06 | 3.77 |  |  |  |  |  |  |
| **STM2586** | - | STM2586 | phage tail assembly-like protein | 11.700 | 5.00 | 7.00E-05 | 12.92 | D | -201 | -215 | tcagGTTGATGATATTTATattg | 4.21 | -7.03 |
| **STM2588** | - | STM2588 | tail fiber-like protein | 5.043 | 5.00 | 4.00E-03 | 36.50 | R | 21 | 7 | ccttGTTAAACGAAAGAGAccgg | 4.32 | -7.1 |
| **STM2589** | S | STM2589 | host specificity protein-J-like | 4.885 | 5.00 | 4.00E-03 | 18.45 | D | -293 | -307 | acggGGTAATAAATGGGAAaggg | 4.13 | -6.98 |
| **STM2590** | S | aSTM2590 | tail assembly protein I-like | 12.030 | 5.00 | 5.00E-05 | 28.59 |  |  |  |  |  |  |
| **STM2591** | M | STM2591 | tail assembly protein K-like | 11.550 | 5.00 | 8.00E-05 | 38.58 | R | -131 | -145 | gaaaGTTTATTAATGGAGAggaa | 5.41 | -7.86 |
| **STM2592** | S | STM2592 | phage tail component L-like protein | 7.102 | 5.00 | 8.00E-04 | 19.14 |  |  |  |  |  |  |
| **STM2593** | S | STM2593 | phage tail component M-like protein | 17.710 | 7.00 | 7.00E-07 | 10.29 |  |  |  |  |  |  |
| **STM2594** | S | STM2594 | phage tail component H-like protein | 4.749 | 5.00 | 5.00E-03 | 12.16 |  |  |  |  |  |  |
| **STM2595** | - | STM0917 | minor tail-like protein | 13.730 | 5.00 | 3.00E-05 | 33.85 |  |  |  |  |  |  |
| **STM2596** | - | STM2596 | minor tail-like protein | 18.830 | 5.00 | 7.00E-06 | 51.36 |  |  |  |  |  |  |
| **STM2597** | - | STM2597 | major tail-like protein | 6.325 | 5.00 | 1.00E-03 | 27.51 |  |  |  |  |  |  |
| **STM2598** | - | STM2598 | hypothetical protein | 11.920 | 5.00 | 7.00E-05 | 33.68 |  |  |  |  |  |  |
| **STM2599** | L | STM2599 | Gifsy-1 prophage; K07496 putative transposase | 7.001 | 5.00 | 8.00E-04 | 5.49 |  |  |  |  |  |  |
| **STM2600** | - | STM2600 | minor tail protein Z-like | 6.210 | 5.00 | 2.00E-03 | 22.94 |  |  |  |  |  |  |
| **STM2601** | - | STM2601 | minor capsid protein FII | 7.045 | 5.00 | 9.00E-04 | 26.69 |  |  |  |  |  |  |
| **STM2602** | - | STM2602 | DNA packaging-like protein | 36.690 | 5.00 | 2.00E-07 | 36.81 |  |  |  |  |  |  |
| **STM2603** | - | STM2603 | phage head-like protein | 4.677 | 5.00 | 5.00E-03 | 10.39 | R | -268 | -282 | ggacGTTAAATACGATAAAggcc | 4.02 | -6.9 |
| **STM2604** | - | STM2604 | phage head-like protein | 8.220 | 5.00 | 4.00E-04 | 21.33 |  |  |  |  |  |  |
| **STM2605** | O | STM2605 | head-tail preconnector-like protein | 5.266 | 5.00 | 3.00E-03 | 42.73 |  |  |  |  |  |  |
| **STM2606** | - | STM2606 | head-tail preconnector-like protein | 5.903 | 5.00 | 2.00E-03 | 13.94 |  |  |  |  |  |  |
| **STM2607** | - | STM2607 | head-to-tail joining-like protein | 9.239 | 7.00 | 6.00E-05 | 4.15 |  |  |  |  |  |  |
| **STM2608** | - | STM2608 | terminase-like large protein | 4.957 | 5.00 | 4.00E-03 | 23.24 |  |  |  |  |  |  |
| **STM2609** | L | STM2609 | DNA packaging-like protein | 21.520 | 5.00 | 3.00E-06 | 24.14 |  |  |  |  |  |  |
| **STM2610** | - | STM2610 | hypothetical protein | 14.100 | 5.00 | 3.00E-05 | 10.76 |  |  |  |  |  |  |
| **STM2611** | - | STM2611 | endopeptidase-like protein | 24.920 | 5.00 | 1.00E-06 | 16.43 |  |  |  |  |  |  |
| **STM2617** | - | STM2617 | antiterminator-like protein | 8.773 | 9.00 | 8.00E-06 | 3.23 |  |  |  |  |  |  |
| **STM2646** | R | yfiD | putative formate acetyltransferase | -14.100 | 8.00 | 6.00E-07 | -4.00 |  |  |  |  |  |  |
| **STM2684** | L | recN | protein used in recombination and DNA repair; K03631 DNA repair protein RecN (Recombination protein N) | 6.289 | 5.00 | 1.00E-03 | 2.63 |  |  |  |  |  |  |
| **STM2708** | R | STM2708 | phage tail-like protein | 3.058 | 6.00 | 2.20E-02 | 4.47 |  |  |  |  |  |  |
| **STM2740** | L | STM2740 | integrase-like protein | -6.850 | 6.00 | 6.00E-04 | -3.25 |  |  |  |  |  |  |
| **STM2750** | G | STM2750 | putative PTS system, glucitol/sorbitol-specific enzyme II [EC:2.7.1.69]; K02782 PTS system, glucitol/sorbitol-specific IIB component; K02783 PTS system, glucitol/sorbitol-specific IIC component | 5.633 | 7.00 | 7.00E-04 | 3.32 | D | -109 | -123 | ggccGTTACCTCAATTCCAgtaa | 4.85 | -7.46 |
| **STM2753** | R | STM2753 | putative dehydrogenase | 5.774 | 9.00 | 2.00E-04 | 2.68 |  |  |  |  |  |  |
| **STM2779** |  | aSTM2779 |  | 5.360 | 8.00 | 6.00E-04 | 3.66 |  |  |  |  |  |  |
| **STM2780** | S | STM2780 | secreted effector protein | 6.158 | 6.00 | 1.00E-03 | 4.22 | D | 13 | -1 | ccgtGTTTATTATTTTAAAtgat | 5.91 | -8.24 |
| **STM2783** | P | nxiA | putative nickel transporter; K07241 high-affinity nickel-transport protein | 14.870 | 6.00 | 3.00E-06 | 3.42 | D | -271 | -285 | tagcGTTAATTAAAAATATagtg | 6.87 | -8.99 |
| **STM2784** | T | tctE | tricarboxylic transport: regulatory protein [EC:2.7.13.3]; K07649 two-component system, OmpR family, sensor histidine kinase TctE | 9.434 | 6.00 | 1.00E-04 | 2.75 |  |  |  |  |  |  |
| **STM2786** | S | STM2786 | tricarboxylic transport; K07795 putative tricarboxylic transport membrane protein | 6.408 | 5.00 | 1.00E-03 | 77.56 | R | -333 | -347 | actcATTAATAAAATGCTTgcag | 4.68 | -7.34 |
| **STM2787** | - | STM2787 | tricarboxylic transport; K07794 putative tricarboxylic transport membrane protein | 11.340 | 5.00 | 9.00E-05 | 90.77 |  |  |  |  |  |  |
| **STM2788** | S | STM2788 | tricarboxylic transport; K07793 putative tricarboxylic transport membrane protein | 5.529 | 5.00 | 3.00E-03 | 20.57 |  |  |  |  |  |  |
| **STM2789** | - | STM2789 | putative cytoplasmic protein | 8.551 | 5.00 | 3.00E-04 | 10.40 | D | -162 | -176 | cattGTTAAATATTTGTTGtttt | 4.51 | -7.22 |
| **STM2790** | R | ygaF | putative sarcosine oxidase-like protein | 7.844 | 7.00 | 1.00E-04 | 6.85 | R | -141 | -155 | gattGTTAATCAACAGGAAtttg | 7.93 | -9.9 |
| **STM2791** | C | gabD | succinate-semialdehyde dehydrogenase I [EC:1.2.1.16]; K00135 succinate-semialdehyde dehydrogenase (NADP+) | 13.760 | 6.00 | 8.00E-06 | 3.40 |  |  |  |  |  |  |
| **STM2792** | E | gabT | 4-aminobutyrate aminotransferase [EC:2.6.1.19 2.6.1.22]; K00823 4-aminobutyrate aminotransferase; K07250 (S)-3-amino-2-methylpropionate transaminase | 22.020 | 6.00 | 9.00E-07 | 9.12 |  |  |  |  |  |  |
| **STM2798** | P | ygaP | putative rhodanese-like sulfurtransferase | 11.950 | 10.00 | 5.00E-07 | 2.62 | R | 49 | 35 | tttaGTTAATTCTAAAATA | 6.62 | -8.8 |
| **STM2802** | S | ygaM | putative inner membrane protein | 5.872 | 6.00 | 1.00E-03 | 2.54 | D | -35 | -49 | cgcgGTTAACATAAACATAattc | 5.87 | -8.21 |
| **STM3019** | I | yqeF | acetyl-CoA acetyltransferase [EC:2.3.1.9]; K00626 acetyl-CoA C-acetyltransferase | 8.369 | 5.00 | 3.00E-04 | 3.49 |  |  |  |  |  |  |
| **STM3022** | E | STM3022 | putative transport protein | 5.818 | 6.00 | 1.00E-03 | 3.30 | D | -284 | -298 | tataGTTGATAAATTCAAAtaat | 4.86 | -7.47 |
| **STM3024** | R | yohM | putative inner membrane protein; K08970 nickel/cobalt exporter | 4.500 | 9.00 | 1.00E-03 | 3.00 |  |  |  |  |  |  |
| **STM3049** | R | yqfA | putative hemolysin | -4.750 | 5.00 | 4.00E-03 | -2.64 | R | -211 | -225 | acatGTTAGCTAAAATAAAttcg | 6.74 | -8.89 |
| **STM3113** | G | nupG | MFS family, nucleoside transport; K03289 MFS transporter, NHS family, nucleoside:H+ symporter | -15.000 | 9.00 | 1.00E-07 | -6.94 | R | 44 | 30 | tgcgGTTAATCATATTCGCggcc | 4.17 | -7 |
| **STM3124** | K | STM3124 | putative response regulator | 3.913 | 7.00 | 5.00E-03 | 3.03 | D | -218 | -232 | ttgtGTTAACCGAATGTAAattg | 9.06 | -10.99 |
| **STM3127** | R | STM3127 | putative cytoplasmic protein | 8.181 | 7.00 | 6.00E-05 | 3.00 | R | -311 | -325 | acagGTTGATTGAGTTTAAaagc | 4.61 | -7.29 |
| **STM3138** | N | STM3138 | putative methyl-accepting chemotaxis protein | -7.880 | 7.00 | 1.00E-04 | -2.75 | D | -41 | -55 | tgcaGTTAATTATTATTCAgcta | 9.54 | -11.51 |
| **STM3157** | Q | yghA | putative oxidoreductase [EC:1.-.-.-] | 10.840 | 10.00 | 1.00E-06 | 2.70 | D | -190 | -204 | gctaGTTAACTCTGTTGTGaata | 4.08 | -6.94 |
| **STM3197** | - | glgS | glycogen biosynthesis, rpoS dependent | -7.600 | 5.00 | 5.00E-04 | -5.21 |  |  |  |  |  |  |
| **STM3210** | L | dnaG | DNA biosynthesis; DNA primase [EC:2.7.7.-]; K02316 DNA primase | 14.510 | 7.00 | 2.00E-06 | 2.61 |  |  |  |  |  |  |
| **STM3216** | N | STM3216 | putative methyl-accepting chemotaxis protein; K05875 methyl-accepting chemotaxis protein II, aspartate sensor receptor | -9.070 | 5.00 | 2.00E-04 | -2.75 | R | -103 | -117 | aaagGTTAACCATTTCTTAttta | 8.5 | -10.42 |
| **STM3217** | T | aer | aerotaxis sensor receptor, senses cellular redox state or proton motive force; K03776 aerotaxis receptor | -10.300 | 6.00 | 5.00E-05 | -3.41 | D | -6 | -20 | atgcGTTAACAATTGGATGcatt | 4.33 | -7.1 |
| **STM3218** | E | oat | putative acetylornithine aminotransferase [EC:2.6.1.82]; K09251 putrescine aminotransferase | 11.240 | 6.00 | 4.00E-05 | 6.10 | R | -62 | -76 | atgcGTTAACAATTGGATGcatt | 4.33 | -7.1 |
| **STM3224** | P | ygjT | putative tellurite resistance protein | 15.500 | 9.00 | 1.00E-07 | 2.64 | D | -35 | -49 | ttatGTAAATCATATGTTAcagg | 7.2 | -9.26 |
| **STM3225** | E | ygjU | putative dicarboxylate permease; K03309 dicarboxylate/amino acid:cation (Na+ or H+) symporter, DAACS family | 16.110 | 6.00 | 3.00E-06 | 5.37 | D | -132 | -146 | tactGTTAATATAAAGTTAtaaa | 8.02 | -9.98 |
| **STM3237** | - | yhaL | putative cytoplasmic protein | -4.020 | 6.00 | 8.00E-03 | -3.04 |  |  |  |  |  |  |
| **STM3238** | S | yhaN | putative inner membrane protein | -8.660 | 5.00 | 2.00E-04 | -3.02 |  |  |  |  |  |  |
| **STM3239** | E | yhaO | putative transport protein | -8.130 | 5.00 | 3.00E-04 | -3.97 |  |  |  |  |  |  |
| **STM3280** | - | deaD | cysteine sulfinate desulfinase; K05592 ATP-dependent RNA helicase DeaD | 28.770 | 6.00 | 1.00E-07 | 3.69 |  |  |  |  |  |  |
| **STM3284** | J | truB | tRNA pseudouridine 5S synthase [EC:5.4.99.12]; K03177 tRNA pseudouridine synthase B | 14.250 | 6.00 | 5.00E-06 | 2.52 |  |  |  |  |  |  |
| **STM3288** | S | yhbC | hypothetical protein; K09748 hypothetical protein | 11.430 | 9.00 | 1.00E-06 | 2.60 |  |  |  |  |  |  |
| **STM3301** | R | yhbZ | putative GTP-binding protein | 8.238 | 7.00 | 8.00E-05 | 3.18 |  |  |  |  |  |  |
| **STM3303** | J | rpmA | 50S ribosomal subunit protein L27; K02899 large subunit ribosomal protein L27 | 15.520 | 9.00 | 1.00E-07 | 2.65 | D | -245 | -259 | agtgGTTCACTGATGTGAAaatt | 4.87 | -7.47 |
| **STM3338** | G | nanT | MFS family, sialic acid transport protein; K03290 MFS transporter, SHS family, sialic acid transporter | -6.940 | 6.00 | 6.00E-04 | -2.88 |  |  |  |  |  |  |
| **STM3384** | J | yhdG | putative TIM-barrel enzyme, possibly dehydrogenase [EC:1.-.-.-]; K05540 tRNA-dihydrouridine synthase B | 15.820 | 5.00 | 1.00E-05 | 4.63 |  |  |  |  |  |  |
| **STM3385** | K | fis | site-specific DNA inversion stimulation factor; K03557 Fis family transcriptional regulator, factor for inversion stimulation protein | 11.300 | 10.00 | 6.00E-07 | 2.52 |  |  |  |  |  |  |
| **STM3458** | R | yheR | putative NAD(P)H oxidoreductase [EC:1.6.99.-] | 15.520 | 8.00 | 2.00E-07 | 4.60 | D | -48 | -62 | gtatGTTCAGACTATGTTAattt | 7.38 | -9.42 |
| **STM3536** | G | glgC | glucose-1-phosphate adenylyltransferase [EC:2.7.7.27]; K00975 glucose-1-phosphate adenylyltransferase | -8.420 | 6.00 | 1.00E-04 | -2.54 |  |  |  |  |  |  |
| **STM3556** | G | ugpA | ABC superfamily (membrane), sn-glycerol 3-phosphate transport protein; K05814 sn-Glycerol 3-phosphate transport system permease protein | 4.962 | 7.00 | 1.00E-03 | 3.20 |  |  |  |  |  |  |
| **STM3576** | P | zntA | P-type ATPase family, Pb/Cd/Zn/Hg transporting ATPase [EC:3.6.3.3 3.6.3.5]; K01532 Cd2+-exporting ATPase; K01534 Zn2+-exporting ATPase | 7.083 | 5.00 | 6.00E-04 | 2.76 | R | -275 | -289 | tccgGTTGATGATAATTTTctca | 4.18 | -7 |
| **STM3577** | N | tcp | methyl-accepting transmembrane citrate/phenol chemoreceptor; K03406 methyl-accepting chemotaxis protein | -6.780 | 5.00 | 8.00E-04 | -4.59 | R | -189 | -203 | gtttGATAAGTAATAGAAAataa | 4.97 | -7.55 |
| **STM3587** | M | yhiI | paral putative membrane protein; K01993 HlyD family secretion protein | -12.300 | 6.00 | 1.00E-05 | -2.82 |  |  |  |  |  |  |
| **STM3599** | R | STM3599 | putative inner membrane protein; K07792 anaerobic C4-dicarboxylate transporter DcuB | -5.230 | 6.00 | 2.00E-03 | -3.38 |  |  |  |  |  |  |
| **STM3600** | G | STM3600 | putative sugar kinase | -7.380 | 5.00 | 6.00E-04 | -4.67 | R | -189 | -203 | cgtcGTTAACGGATGCTGAcgcg | 4.7 | -7.36 |
| **STM3601** | M | STM3601 | putative phosphosugar isomerase | -15.400 | 7.00 | 1.00E-06 | -3.89 | R | -104 | -118 | tacgGTAAACCGTATTTTAtcgc | 4.26 | -7.05 |
| **STM3604** | - | STM3604 | putative inner membrane protein | -7.560 | 6.00 | 3.00E-04 | -3.04 | D | -132 | -146 | ccgcGTTGACAGAATGAAAaact | 5.22 | -7.72 |
| **STM3611** | T | yhjH | hypothetical protein | -7.490 | 5.00 | 6.00E-04 | -5.62 | D | -129 | -143 | agtgGTCAATAATATTAAAacat | 5.77 | -8.13 |
| **STM3625** | E | yhjV | putative transport protein | 7.089 | 7.00 | 3.00E-04 | 5.02 | R | -316 | -330 | tactGTTATTTAATGGTAAtgtg | 6.19 | -8.45 |
| **STM3626** | E | dppF | ABC superfamily (atp_bind), dipeptide transport protein; K02032 peptide/nickel transport system ATP-binding protein | 8.379 | 6.00 | 2.00E-04 | 4.32 |  |  |  |  |  |  |
| **STM3627** | E | dppD | ABC superfamily (atp_bind), dipeptide transport protein; K02031 peptide/nickel transport system ATP-binding protein | 19.100 | 6.00 | 9.00E-07 | 5.15 |  |  |  |  |  |  |
| **STM3628** | E | dppC | ABC superfamily (membrane), dipeptide transport protein 2; K02034 peptide/nickel transport system permease protein | 5.018 | 6.00 | 2.00E-03 | 3.18 |  |  |  |  |  |  |
| **STM3629** | E | dppB | ABC superfamily (membrane), dipeptide transport protein 1; K02033 peptide/nickel transport system permease protein | 15.490 | 10.00 | 4.00E-08 | 5.12 |  |  |  |  |  |  |
| **STM3630** | E | dppA | ABC superfamily (peri_perm), dipeptide transport protein; K02035 peptide/nickel transport system substrate-binding protein | 25.800 | 10.00 | 4.00E-10 | 3.14 | R | -111 | -125 | atctGTCAATAGAATGTCAaaac | 5.65 | -8.04 |
| **STM3651** | K | STM3651 | putative acetyltransferase | 6.317 | 8.00 | 2.00E-04 | 3.03 | D | -17 | -31 | tcttGTAAAGCAAATGCTAtaca | 4.59 | -7.28 |
| **STM3690** | - | STM3690 | putative inner membrane lipoprotein | -4.850 | 5.00 | 4.00E-03 | -3.19 | D | -257 | -271 | acagGTTAACTATTTATATtata | 5.56 | -7.97 |
| **STM3692** | C | lldP | LctP transporter, L-lactate permease; K03303 lactate transporter, LctP family | 5.245 | 5.00 | 3.00E-03 | 12.78 | R | -128 | -142 | tagaGTTAATTTAATGAAAtgtg | 8.53 | -10.45 |
| **STM3693** | K | lldR | putative transcriptional regulator | 6.523 | 5.00 | 1.00E-03 | 80.00 |  |  |  |  |  |  |
| **STM3694** | C | lldD | L-lactate dehydrogenase [EC:1.1.2.3]; K00101 L-lactate dehydrogenase (cytochrome) | 4.968 | 5.00 | 4.00E-03 | 23.12 |  |  |  |  |  |  |
| **STM3695** | J | yibK | putative tRNA/rRNA methyltransferase [EC:2.1.1.-]; K03216 RNA methyltransferase, TrmH family, group 2 | 11.870 | 9.00 | 9.00E-07 | 4.22 |  |  |  |  |  |  |
| **STM3704** | G | pmgI | phosphoglyceromutase [EC:5.4.2.1]; K01834 phosphoglycerate mutase | 13.390 | 6.00 | 2.00E-05 | 3.14 |  |  |  |  |  |  |
| **STM3761** | Q | slsA | putative inner membrane protein | -8.110 | 5.00 | 4.00E-04 | -3.15 |  |  |  |  |  |  |
| **STM3763** | P | mgtB | Mg2+ transport protein [EC:3.6.3.2]; K01531 Mg2+-importing ATPase | 10.340 | 6.00 | 6.00E-05 | 2.83 |  |  |  |  |  |  |
| **STM3764** | S | mgtC | Mg2+ transport protein; K07507 putative Mg2+ transporter-C (MgtC) family protein | 22.420 | 7.00 | 7.00E-08 | 5.32 |  |  |  |  |  |  |
| **STM3784** | G | STM3784 | PTS system, galactitol-specific IIA component, putative [EC:2.7.1.69]; K02773 PTS system, galactitol-specific IIA component | -7.200 | 8.00 | 1.00E-04 | -3.16 |  |  |  |  |  |  |
| **STM3803** | R | yidF | putative cytoplasmic protein | -4.800 | 5.00 | 4.00E-03 | -3.83 |  |  |  |  |  |  |
| **STM3808** | - | ibpB | small heat shock protein; K04081 molecular chaperone IbpB | 8.793 | 7.00 | 4.00E-05 | 2.74 |  |  |  |  |  |  |
| **STM3855** | P | pstA | ABC superfamily (membrane), high-affinity phosphate transporter; K02038 phosphate transport system permease protein | 15.730 | 6.00 | 2.00E-06 | 3.86 |  |  |  |  |  |  |
| **STM3856** | P | pstC | ABC superfamily (membrane), high-affinity phosphate transporter; K02037 phosphate transport system permease protein | 10.720 | 6.00 | 4.00E-05 | 4.23 | R | -156 | -170 | attcGTTAATTAATACAGCgcct | 5.17 | -7.69 |
| **STM3857** | P | pstS | ABC superfamily (bind_prot), high-affinity phosphate transporter; K02040 phosphate transport system substrate-binding protein | 28.350 | 7.00 | 4.00E-08 | 9.85 |  |  |  |  |  |  |
| **STM3956** | Q | yigI | putative protein PaaI, possibly involved in aromatic compounds catabolism | 12.900 | 10.00 | 2.00E-07 | 3.01 | R | -284 | -298 | attaGTTAATGAAATGTTGatat | 7.9 | -9.88 |
| **STM3968** | F | udp | uridine phosphorylase [EC:2.4.2.3]; K00757 uridine phosphorylase | -5.070 | 5.00 | 4.00E-03 | -5.00 | R | -155 | -169 | tgatGTTCATCACAATAAAtaat | 6.06 | -8.35 |
| **STM3982** | I | fadA | 3-ketoacyl-CoA thiolase; (thiolase I, acetyl-CoA transferase), in complex with FadB catalyzes EC 2.3.1.16 reaction [EC:2.3.1.16]; K00632 acetyl-CoA acyltransferase | 14.770 | 5.00 | 2.00E-05 | 6.28 | R | 2 | -12 | cttcGTTAATCATCGGGATcatc | 4.53 | -7.24 |
| **STM3983** | I | fadB | enoyl-CoA hydratase / dodecenoyl-CoA delta-isomerase / 3-hydroxyacyl-CoA dehydrogenase / 3-hydroxybutyryl-CoA epimerase [EC:4.2.1.17 5.3.3.8 1.1.1.35 5.1.2.3]; K00022 3-hydroxyacyl-CoA dehydrogenase; K01692 enoyl-CoA hydratase; K01782 3-hydrox • • • | 9.780 | 5.00 | 2.00E-04 | 11.16 | R | -223 | -237 | ttgtGTTAAAAAAATGCAAataa | 6.8 | -8.94 |
| **STM4014** | - | STM4014 | putative periplasmic protein | 4.429 | 9.00 | 2.00E-03 | 2.70 |  |  |  |  |  |  |
| **STM4044** | C | STM4044 | putative alcohol dehydrogenase | -6.000 | 5.00 | 2.00E-03 | -3.48 |  |  |  |  |  |  |
| **STM4045** | G | rhaD | rhamnulose-1-phosphate aldolase [EC:4.1.2.19]; K01629 rhamnulose-1-phosphate aldolase | -4.120 | 6.00 | 7.00E-03 | -2.59 |  |  |  |  |  |  |
| **STM4060** | U | cpxP | periplasmic repressor of cpx regulon by interaction with CpxA, rescue from transitory stresses; K06006 periplasmic protein CpxP | 7.899 | 6.00 | 2.00E-04 | 3.24 | D | 2 | -12 | tgacGTCAAGCAAAAGTAAatcg | 5.02 | -7.58 |
| **STM4063** | P | sbp | ABC superfamily (bind_prot), sulfate transport protein; K02048 sulfate transport system substrate-binding protein | 3.275 | 6.00 | 1.80E-02 | 3.03 |  |  |  |  |  |  |
| **STM4071** | S | STM4071 | putative mannose-6-phosphate isomerase | -12.000 | 5.00 | 6.00E-05 | -10.00 |  |  |  |  |  |  |
| **STM4072** | G | ydeV | putative sugar kinase | -11.700 | 5.00 | 7.00E-05 | -9.90 | D | -142 | -156 | gcgcGGTAACTATATCAATgcac | 4.51 | -7.22 |
| **STM4073** | K | ydeW | putative transcriptional repressor | -8.160 | 5.00 | 3.00E-04 | -7.87 | D | -258 | -272 | ttgtGTTAATGATTTAGAAatgt | 4.82 | -7.44 |
| **STM4074** | G | ego | putative ABC-type sugar, aldose transport system, ATPase component [EC:3.6.3.17]; K02056 simple sugar transport system ATP-binding protein | -22.600 | 5.00 | 2.00E-06 | -17.86 | R | -74 | -88 | ttgtGTTAATGATTTAGAAatgt | 4.82 | -7.44 |
| **STM4075** | G | ydeY | putative ABC superfamily (membrane), sugar transport protein; K02057 simple sugar transport system permease protein | -19.700 | 5.00 | 3.00E-06 | -16.67 |  |  |  |  |  |  |
| **STM4076** | G | ydeZ | putative ABC superfamily (membrane), sugar transport protein; K02057 simple sugar transport system permease protein | -6.910 | 5.00 | 9.00E-04 | -10.42 |  |  |  |  |  |  |
| **STM4077** | G | yneA | putative ABC superfamily (peri_perm), sugar transport protein; K02058 simple sugar transport system substrate-binding protein | -15.300 | 5.00 | 1.00E-05 | -9.43 |  |  |  |  |  |  |
| **STM4078** | G | yneB | putative fructose-1,6-bisphosphate aldolase [EC:4.1.2.-]; K08321 putative fructose-bisphosphate aldolase | -10.700 | 5.00 | 9.00E-05 | -5.32 |  |  |  |  |  |  |
| **STM4079** | - | yneC | putative inner membrane protein | -6.050 | 6.00 | 1.00E-03 | -4.44 |  |  |  |  |  |  |
| **STM4080** | G | STM4080 | ribulose-phosphate 3-epimerase | -10.400 | 10.00 | 1.00E-06 | -3.05 |  |  |  |  |  |  |
| **STM4085** | G | glpX | unknown function in glycerol metabolism [EC:3.1.3.11]; K02446 fructose-1,6-bisphosphatase II | 10.630 | 9.00 | 3.00E-06 | 2.57 |  |  |  |  |  |  |
| **STM4094** | K | cytR | transcriptional repressor (GalR/LacI family); K05499 LacI family transcriptional regulator, repressor for deo operon, udp, cdd, tsx, nupC, and nupG | -16.700 | 9.00 | 3.00E-08 | -3.97 |  |  |  |  |  |  |
| **STM4108** | C | gldA | glycerol dehydrogenase [EC:1.1.1.6]; K00005 glycerol dehydrogenase | -9.430 | 5.00 | 2.00E-04 | -2.86 |  |  |  |  |  |  |
| **STM4112** | G | frwC | PTS system fructose-like IIC component; K02770 PTS system, fructose-specific IIC component | -5.880 | 6.00 | 1.00E-03 | -2.52 |  |  |  |  |  |  |
| **STM4126** | C | udhA | soluble pyridine nucleotide transhydrogenase [EC:1.6.1.1]; K00322 NAD(P) transhydrogenase | 7.666 | 7.00 | 1.00E-04 | 3.10 |  |  |  |  |  |  |
| **STM4158** | - | STM4158 | putative cytoplasmic protein | 5.448 | 5.00 | 3.00E-03 | 3.72 |  |  |  |  |  |  |
| **STM4198** | - | STM4198 | putative cytoplasmic protein | 3.610 | 8.00 | 6.00E-03 | 2.62 |  |  |  |  |  |  |
| **STM4238** | V | dinF | DNA-damage-inducible protein F, induced by UV and mitomycin C; SOS, lexA regulon; K03327 multidrug resistance protein, MATE family | 8.962 | 7.00 | 7.00E-05 | 3.01 | D | -275 | -289 | ttcgTTTAACTCCTTGTCAggct | 4.04 | -6.91 |
| **STM4273** | R | yjcG | putative SSS family transport protein; K03307 solute:Na+ symporter, SSS family | 6.908 | 6.00 | 6.00E-04 | 2.94 |  |  |  |  |  |  |
| **STM4274** | S | yjcH | putative inner membrane protein | 3.933 | 6.00 | 7.00E-03 | 4.28 | D | -217 | -231 | tcaaGTTCACTATGTGGCTgggg | 6.09 | -8.37 |
| **STM4275** | I | acs | acetyl-coenzyme A synthetase [EC:6.2.1.1]; K01895 acetyl-CoA synthetase | 12.320 | 6.00 | 3.00E-05 | 12.08 | R | -264 | -278 | ggatGTTAATAATATGTCGcata | 7.15 | -9.22 |
| **STM4277** | P | nrfA | nitrite reductase periplasmic cytochrome c552 [EC:1.7.2.2]; K03385 formate-dependent nitrite reductase, periplasmic cytochrome c552 subunit | -21.300 | 9.00 | 1.00E-08 | -3.51 | R | -152 | -166 | tgtcTTTAATCAATTGTAAgtgt | 5.69 | -8.07 |
| **STM4278** | - | nrfB | formate-dependent nitrite reductase; K04013 formate-dependent nitrite reductase, penta-haeme cytochrome c | -6.230 | 7.00 | 5.00E-04 | -2.69 |  |  |  |  |  |  |
| **STM4295** | K | adiY | transcriptional activator of adiA (AraC/XylS family); K03755 AraC family transcriptional regulator, transcriptional activator of adiA | 14.490 | 6.00 | 1.00E-05 | 3.63 |  |  |  |  |  |  |
| **STM4301** | R | dcuB | Dcu family, anaerobic C4-dicarboxylate transporter; K07792 anaerobic C4-dicarboxylate transporter DcuB | 13.270 | 8.00 | 2.00E-06 | 3.17 | R | -27 | -41 | aggcGTTTATTATTTGATTtttg | 5.53 | -7.95 |
| **STM4314** | K | STM4314 | putative regulatory protein | 4.920 | 9.00 | 9.00E-04 | 2.58 |  |  |  |  |  |  |
| **STM4320** | K | STM4320 | putative regulatory protein | 3.992 | 8.00 | 4.00E-03 | 2.52 |  |  |  |  |  |  |
| **STM4345** | E | yjeM | putative APC family, amino-acid transport protein | 4.544 | 10.00 | 1.00E-03 | 2.53 |  |  |  |  |  |  |
| **STM4355** | - | yjeS | putative FeS protein | 12.910 | 6.00 | 1.00E-05 | 2.55 |  |  |  |  |  |  |
| **STM4377** | I | aidB | putative acyl-CoA dehydrogenase; adaptive response (transcription activated by Ada); K09456 putative acyl-CoA dehydrogenase | -7.360 | 8.00 | 9.00E-05 | -2.81 | D | -212 | -226 | gccgGTTAATCATACTTTTgtga | 5.1 | -7.63 |
| **STM4378** | - | yjfN | putative inner membrane protein | -6.390 | 5.00 | 1.00E-03 | -6.25 |  |  |  |  |  |  |
| **STM4384** | G | sgaB | putative PTS enzyme IIsga subunit [EC:2.7.1.69]; K02822 PTS system, unknown pentitol phosphotransferase enzyme IIB component | -6.060 | 8.00 | 3.00E-04 | -2.69 |  |  |  |  |  |  |
| **STM4385** | G | ptxA | putative PTS enzyme IIsga subunit [EC:2.7.1.69]; K02821 PTS system, unknown pentitol phosphotransferase enzyme IIA component | -3.940 | 5.00 | 9.00E-03 | -2.64 |  |  |  |  |  |  |
| **STM4398** | E | cycA | APC family, D-alanine/D-serine/glycine transport protein; K03293 amino acid transporter, AAT family | 8.287 | 5.00 | 3.00E-04 | 3.81 | R | -154 | -168 | caatGTTAATTTTATGTTTaaaa | 7.4 | -9.44 |
| **STM4400** | G | ytfF | putative cationic amino acid transporter | 9.733 | 7.00 | 3.00E-05 | 2.73 | R | -225 | -239 | ataaATTAACTAAATTTTAacat | 8.09 | -10.04 |
| **STM4408** | O | msrA | peptide methionine sulfoxide reductase [EC:1.8.4.11]; K07304 peptide-methionine (S)-S-oxide reductase | 12.600 | 6.00 | 9.00E-06 | 3.76 | R | -151 | -165 | aattGTTGATTTTATGTTAagcc | 5.37 | -7.83 |
| **STM4421** | C | STM4421 | putative NAD-dependent aldehyde dehydrogenase [EC:1.2.1.27]; K00140 methylmalonate-semialdehyde dehydrogenase | -13.300 | 10.00 | 1.00E-07 | -2.51 | D | -87 | -101 | ataaGTTAATTATATTTTTgatt | 10.28 | -12.39 |
| **STM4459** | F | pyrI | aspartate carbamoyltransferase, regulatory subunit (allosteric regulation) [EC:2.1.3.2]; K00610 aspartate carbamoyltransferase regulatory chain | -7.990 | 5.00 | 4.00E-04 | -3.58 |  |  |  |  |  |  |
| **STM4462** |  | yjgG |  | -10.800 | 5.00 | 9.00E-05 | -21.28 |  |  |  |  |  |  |
| **STM4463** | E | STM4463 | putative arginine repressor | -29.100 | 5.00 | 8.00E-07 | -83.33 |  |  |  |  |  |  |
| **STM4464** | S | STM4464 | putative arginine repressor | -6.240 | 5.00 | 2.00E-03 | -55.55 |  |  |  |  |  |  |
| **STM4465** | E | STM4465 | ornithine carbamoyltransferase [EC:2.1.3.3]; K00611 ornithine carbamoyltransferase | -5.990 | 5.00 | 2.00E-03 | -20.83 | D | -304 | -318 | aatgGTTATTTCACTGAAAaatc | 5.26 | -7.75 |
| **STM4466** | E | STM4466 | carbamate kinase [EC:2.7.2.2]; K00926 carbamate kinase | -6.000 | 5.00 | 2.00E-03 | -20.41 | D | -15 | -29 | gctgGTTCATGCTCTTGAAacag | 4.38 | -7.14 |
| **STM4467** | E | STM4467 | arginine deiminase [EC:3.5.3.6]; K01478 arginine deiminase | -6.060 | 5.00 | 2.00E-03 | -23.81 | R | -30 | -44 | tattGTTAATTATTTGTTTgctg | 9.98 | -12.01 |
| **STM4469** | E | argI | ornithine carbamoyltransferase 1 [EC:2.1.3.3]; K00611 ornithine carbamoyltransferase | -11.000 | 6.00 | 3.00E-05 | -8.26 | D | -263 | -277 | gaatTTTAATTCAATGAGAggcc | 6.34 | -8.56 |
| **STM4510** | M | STM4510 | putative aspartate racemase | -8.290 | 5.00 | 4.00E-04 | -5.46 |  |  |  |  |  |  |
| **STM4511** | K | yjiE | putative transcriptional regulator | -6.570 | 5.00 | 1.00E-03 | -2.87 | D | 39 | 25 | gctgGTTAACCACTACGGAttca | 5.14 | -7.66 |
| **STM4512** | - | iadA | isoaspartyl dipeptidase [EC:3.4.19.5]; K01305 beta-aspartyl-peptidase | -4.100 | 5.00 | 8.00E-03 | -2.67 |  |  |  |  |  |  |
| **STM4513** | S | yjiG | putative permease | -10.300 | 6.00 | 7.00E-05 | -2.96 |  |  |  |  |  |  |
| **STM4526** | V | hsdR | endonuclease R, host restriction [EC:3.1.21.3]; K01153 type I restriction enzyme, R subunit | -4.930 | 5.00 | 4.00E-03 | -3.45 | D | -70 | -84 | actgGTTCAATAAATTTGTcata | 5.22 | -7.72 |
| **STM4558** | R | rimI | modification of 30S ribosomal subunit protein S18; acetylation of N-terminal alanine [EC:2.3.1.128]; K03789 ribosomal-protein-alanine N-acetyltransferase | 8.149 | 6.00 | 2.00E-04 | 2.75 |  |  |  |  |  |  |
| **STM4562** | - | STM4562 | putative inner membrane protein | 13.430 | 9.00 | 3.00E-07 | 2.54 |  |  |  |  |  |  |
| **STM4567** | F | deoC | 2-deoxyribose-5-phosphate aldolase [EC:4.1.2.4]; K01619 deoxyribose-phosphate aldolase | -11.700 | 6.00 | 3.00E-05 | -3.02 | R | -209 | -223 | cacaGTTAATGAAAACTACgtac | 5.49 | -7.92 |
| **STM4568** | F | deoA | thymidine phosphorylase [EC:2.4.2.4]; K00758 thymidine phosphorylase | -13.500 | 7.00 | 2.00E-06 | -3.27 |  |  |  |  |  |  |
| **STM4599** | - | yjjY | putative inner membrane protein | -10.700 | 6.00 | 3.00E-05 | -6.33 |  |  |  |  |  |  |

^a^Location of the open reading frame (ORF) in the S. Typhimurium LT2 genome.

^b^Functional category assigned to the gene by the National Center for Biotechnology Information, Cluster of Orthologous Genes (COGs). The designations of functional categories are as follows:

C, energy production and conversion, D, cell cycle control and mitosis, E, amino acid metabolism and transport, F, nucleotide metabolism and transport, G, carbohydrate metabolism and transport, H, coenzyme metabolism and transport, I, lipid metabolism and transport, J, translation, K, transcription, L, replication, recombination, and repair, M, cell wall/membrane/envelope biogenesis,

N, Cell motility, O, post-translational modification, protein turnover, chaperone functions, P, inorganic ion transport and metabolism, Q, secondary metabolites biosynthesis, transport, and catabolism,

R, general functional prediction only (typically, prediction of biochemical activity), S, function unknown, T, signal transduction mechanisms, U, intracellular trafficking and secretion,

V, defense mechanisms, -, not in COGs.

^c^Respective gene name or symbol.

^d^Functional classification according to the KEGG (Kyoto Encyclopedia of Genes and Genomes) database.

^e^The numerical value of t for the t test (statistical method).

^f^The degrees of freedom employed for the analysis of each gene.

^g^The probability associated with the t test for each gene.

^h^Ratio between the expression level of the *arcA* mutant versus the wild-type.

^i^The strand on which the motif has been localized. R, reverse; D, direct; NA, not available in the Regulatory Sequence Analysis Tools (RSAT) database (the locus identity was not recognized), and a blank cell indicates that no motif was present.

^j^The starting position of the putative motif. The positions are relative to the starting ATG.

^k^The ending position of the putative motif. The positions are relative to the starting ATG.

^l^The sequence of the HIGHEST RANKING putative motif (capitalized letters) and 4 base pairs (bps) flanking either side of the region (lower case letters). A blank cell indicates that no motif was present. All of the sequences are reported from the 5' to the 3' end of the ORF (Open Reading Frame) analyzed.

^m^The score indicating the similarity of the motif to the information matrix. The cutoff used was a score higher than 4.00 or a ln(P) lower than -6.5.

^n^The natural logarithm of the probability that the putative motif is randomly similar to the information matrix. The cutoff used was a score higher than 4.00 or a ln(P) lower than -6.5.

**Table S2. Comparison of genes regulated by Fnr and ArcA in *S*. Typhimurium under anaerobiosis**

| **Locus^a^** | **Category^b^** | **Name^c^** | **STM Gene Function^d^** | **ArcA Ratio^e^** | **ArcA**  **Motif^f^** | **Fnr Ratio^g^** | **Fnr Motif^h^** |
| --- | --- | --- | --- | --- | --- | --- | --- |
| **PSLT019** |  | pefB | plasmid-encoded fimbriae; regulation | -2.74 | + | -4.59 | + |
| **STM0002** | E | thrA | aspartokinase I , bifunctional enxyme N-terminal is aspartokinaseI and C-terminal is homoserine dehydrogenase I | -3.79 | + | -3.33 | + |
| **STM0439** | H | cyoE | protohaeme IX farnesyltransferase (haeme O biosynthesis) [EC:2.5.1.-]; K02301 protoheme IX farnesyltransferase | 4.79 | - | 7.71 | + |
| **STM0440** | C | cyoD | cytochrome o ubiquinol oxidase subunit IV; K02300 cytochrome o ubiquinol oxidase operon protein cyoD | 5.76 | + | 7.05 | + |
| **STM0441** | C | cyoC | cytochrome o ubiquinol oxidase subunit III [EC:1.10.3.-]; K02299 cytochrome o ubiquinol oxidase subunit III | 5.47 | - | 7.10 | - |
| **STM0442** | C | cyoB | cytochrome o ubiquinol oxidase subunit I [EC:1.10.3.-]; K02298 cytochrome o ubiquinol oxidase subunit I | 3.58 | + | 5.05 | + |
| **STM0443** | C | cyoA | cytochrome o ubiquinol oxidase subunit II [EC:1.10.3.-]; K02297 cytochrome o ubiquinol oxidase subunit II | 3.68 | + | 4.51 | + |
| **STM0467** |  | ffs | signal recognition particle, RNA component; K01983 component of ribonucleoprotein particle | -3.35 | - | -4.45 | - |
| **STM0650** | G | STM0650 | putative hydrolase C-terminus [EC:4.2.1.7]; K01685 altronate hydrolase | -8.13 | - | -4.56 | + |
| **STM0662** | E | gltL | ABC superfamily (atp_bind), glutamate/aspartate transporter [EC:3.6.3.-]; K10004 glutamate/aspartate transport system ATP-binding protein | 12.11 | - | 4.39 | + |
| **STM0663** | E | gltK | ABC superfamily (membrane), glutamate/aspartate transporter; K10002 glutamate/aspartate transport system permease protein | 7.23 | + | 2.81 | - |
| **STM0664** | E | gltJ | ABC superfamily (membrane), glutamate/aspartate transporter; K10003 glutamate/aspartate transport system permease protein | 8.47 | - | 2.67 | + |
| **STM0665** | E | gltI | ABC superfamily (bind_prot), glutamate/aspartate transporter; K10001 glutamate/aspartate transport system substrate-binding protein | 4.52 | + | 4.24 | + |
| **STM0699** | R | STM0699 | putative cytoplasmic protein | -4.88 | - | -4.22 | + |
| **STM0738** | C | sucC | succinyl-CoA synthetase, beta subunit [EC:6.2.1.5]; K01903 succinyl-CoA synthetase beta chain | 2.84 | - | 4.03 | + |
| **STM0739** | C | sucD | succinyl-CoA synthetase, alpha subunit [EC:6.2.1.5]; K01902 succinyl-CoA synthetase alpha chain | 3.65 | + | 4.66 | - |
| **STM0790** |  | hutU | pseudogene; frameshift relative to Pseudomonas putida urocanate hydratase; HUTU (SW:P25080) | 5.72 | - | 5.44 | - |
| **STM0791** | E | hutH | histidine ammonia lyase [EC:4.3.1.3]; K01745 histidine ammonia-lyase | 6.57 | + | 4.55 | - |
| **STM0907** | R | aSTM0907 | Fels-1 prophage; putative chitinase; K03791 putative chitinase | -2.79 | - | -3.12 | - |
| **STM1123** | S | STM1123 | putative periplasmic protein | 2.91 | + | 3.13 | - |
| **STM1124** |  | putA | bifunctional in plasma membrane proline dehydrogenase and pyrroline-5-carboxylate dehydrogenase OR in cytoplasm a transcriptional repressor [EC:1.5.1.12 1.5.99.8]; K00294 1-pyrroline-5-carboxylate dehydrogenase; K00318 proline dehydrogenase | 4.49 | - | 7.57 | - |
| **STM1125** | E | putP | SSS family, major sodium/proline symporter; K03307 solute:Na+ symporter, SSS family | 8.50 | + | 7.16 | + |
| **STM1129** | G | STM1129 | putative N-acylglucosamine-6-phosphate 2-epimerase [EC:5.1.3.9]; K01788 N-acylglucosamine-6-phosphate 2-epimerase | -3.77 | - | -6.50 | - |
| **STM1130** | S | STM1130 | putative inner membrane protein | -3.50 | + | -11.85 | - |
| **STM1131** | - | STM1131 | putative outer membrane protein | -3.15 | - | -4.67 | + |
| **STM1132** | G | STM1132 | putative sialic acid transporter | -3.21 | - | -6.20 | + |
| **STM1133** | R | STM1133 | putative dehydrogenase | -2.59 | - | -5.56 | + |
| **STM1138** | - | ycdZ | putative inner membrane protein | -13.16 | + | -11.00 | + |
| **STM1171** | N | flgN | flagellar biosynthesis: belived to be export chaperone for FlgK and FlgL; K02399 flagella synthesis protein FlgN | -5.43 | + | -7.85 | - |
| **STM1172** | K | flgM | anegative regulator of flagellin synthesis (anti-sigma factor); K02398 negative regulator of flagellin synthesis FlgM | -6.13 | + | -5.75 | + |
| **STM1183** | N | flgK | flagellar biosynthesis, hook-filament junction protein 1; K02396 flagellar hook-associated protein 1 FlgK | -2.75 | + | -5.67 | - |
| **STM1184** | N | flgL | flagellar biosynthesis; hook-filament junction protein; K02397 flagellar hook-associated protein 3 FlgL | -2.90 | - | -4.18 | - |
| **STM1300** | - | STM1300 | putative periplasmic protein | -5.05 | - | -4.83 | + |
| **STM1301** | L | STM1301 | putative mutator MutT protein [EC:3.6.1.-]; K08320 CTP pyrophosphohydrolase | -3.08 | - | -3.36 | - |
| **STM1538** | C | STM1538 | hydrogenase large chain [EC:1.12.99.6]; K06281 hydrogenase large subunit | 3.87 | - | 3.96 | - |
| **STM1539** | C | STM1539 | hydrogenase small chain [EC:1.12.99.6]; K06282 hydrogenase small subunit | 3.32 | + | 3.53 | - |

| **STM1795** | E | STM1795 | putative glutamic dehyrogenase-like protein [EC:1.4.1.3]; K00261 glutamate dehydrogenase (NAD(P)+) | 4.04 | + | 5.33 | + |
| --- | --- | --- | --- | --- | --- | --- | --- |
| **STM1803** | E | dadA | D-amino acid dehydrogenase subunit [EC:1.4.99.1]; K00285 D-amino-acid dehydrogenase | 10.52 | + | 3.16 | + |
| **STM1916** | T | cheY | chemotaxis regulator, transmits chemoreceptor signals to flagelllar motor components; K03413 two-component system, chemotaxis family, response regulator CheY | -3.11 | - | -3.74 | + |
| **STM1917** | N | cheB | protein-glutamate methylesterase [EC:3.1.1.61]; K03412 protein-glutamate methylesterase, two-component system, chemotaxis family, response regulator CheB | -3.10 | + | -4.33 | - |
| **STM1918** | N | cheR | chemotaxis protein methyltransferase [EC:2.1.1.80]; K00575 chemotaxis protein methyltransferase CheR | -3.01 | - | -3.32 | - |
| **STM1919** | N | cheM | methyl accepting chemotaxis protein II, aspartate sensor-receptor; K05875 methyl-accepting chemotaxis protein II, aspartate sensor receptor | -3.83 | - | -8.31 | - |
| **STM1920** | N | cheW | purine-binding chemotaxis protein; regulation; K03408 purine-binding chemotaxis protein CheW | -2.94 | - | -4.31 | + |
| **STM1922** | N | motB | Chemotaxis MotB protein; K02557 chemotaxis MotB protein | -3.16 | - | -7.00 | + |
| **STM1923** | N | motA | proton conductor component of motor, torque generator; K02556 chemotaxis MotA protein | -3.95 | - | -10.82 | - |
| **STM1955** | - | fliZ | putative regulator of FliA; K02425 FliZ protein | -3.31 | + | -6.25 | + |
| **STM1956** | K | fliA | sigma F (sigma 28) factor of RNA polymerase, transcription of late flagellar genes (class 3a and 3b operons); K02405 RNA polymerase sigma factor for flagellar operon FliA | -2.58 | + | -5.96 | + |
| **STM1961** | O | fliS | flagellar biosynthesis; repressor of class 3a and 3b operons (RflA activity); K02422 flagellar protein FliS | -2.59 | - | -5.10 | + |
| **STM2183** | F | cdd | cytidine deaminase [EC:3.5.4.5]; K01489 cytidine deaminase | -13.16 | - | -7.71 | + |
| **STM2186** | E | STM2186 | putative NADPH-dependent glutamate synthase beta chain or related oxidoreductase | -4.00 | + | -4.18 | + |
| **STM2187** | F | yeiA | dihydropyrimidine dehydrogenase | -3.92 | + | -4.05 | - |
| **STM2314** | T | STM2314 | putative chemotaxis signal transduction protein [EC:2.7.3.-]; K03415 two-component system, chemotaxis family, response regulator CheV | -5.75 | _ | -6.99 | - |
| **STM2315** | R | yfbK | putative von Willebrand factor, vWF type A domain | -2.93 | + | -2.84 | + |
| **STM2340** | G | STM2340 | putative transketolase [EC:2.2.1.1]; K00615 transketolase | 4.24 | + | 6.60 | + |
| **STM2341** | G | STM2341 | putative transketolase [EC:2.2.1.1]; K00615 transketolase | 4.28 | + | 6.47 | - |

| **STM2342** | S | STM2342 | PTS system, unknown pentitol phosphotransferase enzyme IIC component; K03475 PTS system, unknown pentitol phosphotransferase enzyme IIC component | 4.45 | + | 5.22 | + |
| --- | --- | --- | --- | --- | --- | --- | --- |
| **STM2343** | G | STM2343 | putative sugar phosphotransferase component IIB [EC:2.7.1.69]; K02822 PTS system, unknown pentitol phosphotransferase enzyme IIB component | 3.48 | - | 3.34 | + |
| **STM2409** | F | nupC | NUP family, nucleoside transport; K03317 concentrative nucleoside transporter, CNT family | -3.11 | + | -3.91 | + |
| **STM2454** | K | eutR | putative regulator ethanolamine operon (AraC/XylS family); K04033 AraC family transcriptional regulator, ethanolamine operon transcriptional activator | -5.32 | - | -5.13 | - |
| **STM2455** | Q | eutK | putative carboxysome structural protein, ethanolamine utilization; K04025 ethanolamine utilization protein EutK | -4.81 | - | -6.77 | + |
| **STM2456** | E | eutL | putative carboxysome structural protein, ethanolamine utilization; K04026 ethanolamine utilization protein EutL | -5.13 | - | -6.68 | - |
| **STM2457** | E | eutC | ethanolamine ammonia-lyase, light chain [EC:4.3.1.7]; K03736 ethanolamine ammonia-lyase small subunit | -4.20 | - | -7.07 | - |
| **STM2458** | E | eutB | ethanolamine ammonia-lyase, heavy chain [EC:4.3.1.7]; K03735 ethanolamine ammonia-lyase large subunit | -3.94 | - | -6.33 | + |
| **STM2459** | E | eutA | CPPZ-55 prophage; chaperonin in ethanolamine utilization; K04019 ethanolamine utilization protein EutA | -4.27 | - | -6.17 | - |
| **STM2460** | E | eutH | putative transport protein, ethanolamine utilization; K04023 ethanolamine transporter | -3.76 | + | -6.29 | - |
| **STM2462** | E | eutJ | putative ethanolamine utilization protein; K04024 ethanolamine utilization protein EutJ | -4.31 | - | -7.30 | - |
| **STM2463** | C | eutE | putative aldehyde oxidoreductase in ethanolamine utilization; K04021 aldehyde dehydrogenase | -3.70 | - | -7.02 | + |
| **STM2464** | Q | eutN | putative detox protein in ethanolamine utilization; K04028 ethanolamine utilization protein EutN | -6.29 | - | -8.00 | + |
| **STM2465** | Q | eutM | putative detox protein in ethanolamine utilization; K04027 ethanolamine utilization protein EutM | -5.07 | - | -11.02 | + |
| **STM2466** | C | eutD | putative phosphotransacetylase in ethanolamine utilization; K04020 ethanolamine utilization protein EutD | -6.13 | - | -10.36 | - |
| **STM2467** | E | eutT | putative cobalamin adenosyltransferase, ethanolamine utilization [EC:2.5.1.17]; K04032 ethanolamine utilization cobalamin adenosyltransferase | -4.35 | - | -6.59 | + |

| **STM2468** | E | eutQ | putative ethanolamine utilization protein; K04030 ethanolamine utilization protein EutQ | -5.05 | - | -7.60 | - |
| --- | --- | --- | --- | --- | --- | --- | --- |
| **STM2469** | E | eutP | putative ethanolamine utilization protein; K04029 ethanolamine utilization protein EutP | -6.85 | + | -9.38 | + |
| **STM2470** | E | eutS | putative carboxysome structural protein, ethanol utilization; K04031 ethanolamine utilization protein EutS | -9.52 | + | -13.7 | + |
| **STM2558** | E | cadB | APC family, lysine/cadaverine transport protein; K03757 cadaverine:lysine antiporter | -3.29 | + | -6.43 | + |
| **STM2646** | R | yfiD | putative formate acetyltransferase | -4.00 | - | -5.08 | + |
| **STM2786** | S | STM2786 | tricarboxylic transport; K07795 putative tricarboxylic transport membrane protein | 77.56 | + | 8.21 | - |
| **STM2787** | - | STM2787 | tricarboxylic transport; K07794 putative tricarboxylic transport membrane protein | 90.77 | - | 6.89 | - |
| **STM2788** | S | STM2788 | tricarboxylic transport; K07793 putative tricarboxylic transport membrane protein | 20.57 | - | 3.46 | + |
| **STM3019** | I | yqeF | acetyl-CoA acetyltransferase [EC:2.3.1.9]; K00626 acetyl-CoA C-acetyltransferase | 3.49 | - | 2.58 | + |
| **STM3138** | N | STM3138 | putative methyl-accepting chemotaxis protein | -2.75 | + | -4.76 | - |
| **STM3216** | N | STM3216 | putative methyl-accepting chemotaxis protein; K05875 methyl-accepting chemotaxis protein II, aspartate sensor receptor | -2.75 | + | -3.37 | + |
| **STM3217** | T | aer | aerotaxis sensor receptor, senses cellular redox state or proton motive force; K03776 aerotaxis receptor | -3.41 | + | -4.77 | + |
| **STM3225** | E | ygjU | putative dicarboxylate permease; K03309 dicarboxylate/amino acid:cation (Na+ or H+) symporter, DAACS family | 5.37 | + | 3.68 | - |
| **STM3238** | S | yhaN | putative inner membrane protein | -3.02 | - | -3.18 | + |
| **STM3338** | G | nanT | MFS family, sialic acid transport protein; K03290 MFS transporter, SHS family, sialic acid transporter | -2.88 | - | -3.02 | - |
| **STM3576** | P | zntA | P-type ATPase family, Pb/Cd/Zn/Hg transporting ATPase [EC:3.6.3.3 3.6.3.5]; K01532 Cd2+-exporting ATPase; K01534 Zn2+-exporting ATPase | 2.76 | + | 2.55 | + |
| **STM3577** | N | tcp | methyl-accepting transmembrane citrate/phenol chemoreceptor; K03406 methyl-accepting chemotaxis protein | -4.59 | + | -5.64 | + |
| **STM3599** | R | STM3599 | putative inner membrane protein; K07792 anaerobic C4-dicarboxylate transporter DcuB | -3.38 | - | -9.14 | + |
| **STM3600** | G | STM3600 | putative sugar kinase | -4.67 | + | -12.63 | + |
| **STM3601** | M | STM3601 | putative phosphosugar isomerase | -3.89 | + | -11.94 | + |
| **STM3611** | T | yhjH | hypothetical protein | -5.62 | + | -7.89 | - |
| **STM3626** | E | dppF | ABC superfamily (atp_bind), dipeptide transport protein; K02032 peptide/nickel transport system ATP-binding protein | 4.32 | - | 5.86 | - |
| **STM3627** | E | dppD | ABC superfamily (atp_bind), dipeptide transport protein; K02031 peptide/nickel transport system ATP-binding protein | 5.15 | - | 7.65 | + |
| **STM3628** | E | dppC | ABC superfamily (membrane), dipeptide transport protein 2; K02034 peptide/nickel transport system permease protein | 3.18 | - | 4.09 | - |
| **STM3629** | E | dppB | ABC superfamily (membrane), dipeptide transport protein 1; K02033 peptide/nickel transport system permease protein | 5.12 | - | 12.45 | + |
| **STM3630** | E | dppA | ABC superfamily (peri_perm), dipeptide transport protein; K02035 peptide/nickel transport system substrate-binding protein | 3.14 | + | 9.90 | - |
| **STM3690** | - | STM3690 | putative inner membrane lipoprotein | -3.19 | + | -5.67 | + |
| **STM3692** | C | lldP | LctP transporter, L-lactate permease; K03303 lactate transporter, LctP family | 12.78 | + | 16.00 | + |
| **STM3693** | K | lldR | putative transcriptional regulator | 80.00 | - | 30.60 | - |
| **STM3694** | C | lldD | L-lactate dehydrogenase [EC:1.1.2.3]; K00101 L-lactate dehydrogenase (cytochrome) | 23.12 | - | 28.33 | - |
| **STM3695** | J | yibK | putative tRNA/rRNA methyltransferase [EC:2.1.1.-]; K03216 RNA methyltransferase, TrmH family, group 2 | 4.22 | - | 2.95 | - |
| **STM3808** | - | ibpB | small heat shock protein; K04081 molecular chaperone IbpB | 2.74 | - | 2.85 | - |
| **STM4078** | G | yneB | putative fructose-1,6-bisphosphate aldolase [EC:4.1.2.-]; K08321 putative fructose-bisphosphate aldolase | -4.44 | - | 2.57 | + |
| **STM4085** | G | glpX | unknown function in glycerol metabolism [EC:3.1.3.11]; K02446 fructose-1,6-bisphosphatase II | 2.57 | - | 3.20 | + |
| **STM4126** | C | udhA | soluble pyridine nucleotide transhydrogenase [EC:1.6.1.1]; K00322 NAD(P) transhydrogenase | 3.10 | - | 3.17 | - |
| **STM4277** | P | nrfA | nitrite reductase periplasmic cytochrome c552 [EC:1.7.2.2]; K03385 formate-dependent nitrite reductase, periplasmic cytochrome c552 subunit | -3.51 | + | -3.09 | + |
| **STM4278** | - | nrfB | formate-dependent nitrite reductase; K04013 formate-dependent nitrite reductase, penta-haeme cytochrome c | -2.69 | - | -3.69 | + |
| **STM4301** | R | dcuB | Dcu family, anaerobic C4-dicarboxylate transporter; K07792 anaerobic C4-dicarboxylate transporter DcuB | 3.17 | + | 3.38 | + |
| **STM4398** | E | cycA | APC family, D-alanine/D-serine/glycine transport protein; K03293 amino acid transporter, AAT family | 3.81 | + | 2.54 | + |
| **STM4462** |  | yjgG | putative cytoplasmic protein | -21.28 | - | -3.63 | - |
| **STM4463** | E | STM4463 | putative arginine repressor | -83.33 | - | -5.52 | - |
| **STM4469** | E | argI | ornithine carbamoyltransferase 1 [EC:2.1.3.3]; K00611 ornithine carbamoyltransferase | -8.26 | + | -3.36 | - |
| **STM4510** | M | STM4510 | putative aspartate racemase | -5.46 | - | -6.35 | + |
| **STM4511** | K | yjiE | putative transcriptional regulator | -2.87 | + | -6.79 | - |
| **STM4512** | - | iadA | isoaspartyl dipeptidase [EC:3.4.19.5]; K01305 beta-aspartyl-peptidase | -2.67 | - | -6.59 | + |
| **STM4513** | S | yjiG | putative permease | -2.96 | - | -8.61 | - |
| **STM4526** | V | hsdR | endonuclease R, host restriction [EC:3.1.21.3]; K01153 type I restriction enzyme, R subunit | -3.45 | + | -3.28 | + |
| **STM4567** | F | deoC | 2-deoxyribose-5-phosphate aldolase [EC:4.1.2.4]; K01619 deoxyribose-phosphate aldolase | -3.02 | + | -2.91 | + |
| **STM4568** | F | deoA | thymidine phosphorylase [EC:2.4.2.4]; K00758 thymidine phosphorylase | -3.27 | - | -2.76 | - |

^a^Location of the open reading frame (ORF) in the S. Typhimurium LT2 genome.

^b^Functional category assigned to the gene by the National Center for Biotechnology Information, Cluster of Orthologous Genes (COGs). The designations of functional categories are as follows:

C, energy production and conversion, D, cell cycle control and mitosis, E, amino acid metabolism and transport, F, nucleotide metabolism and transport, G, carbohydrate metabolism and transport, H, coenzyme metabolism and transport, I, lipid metabolism and transport, J, translation, K, transcription, L, replication, recombination, and repair, M, cell wall/membrane/envelope biogenesis,

N, Cell motility, O, post-translational modification, protein turnover, chaperone functions, P, inorganic ion transport and metabolism, Q, secondary metabolites biosynthesis, transport, and catabolism,

R, general functional prediction only (typically, prediction of biochemical activity), S, function unknown, T, signal transduction mechanisms, U, intracellular trafficking and secretion,

V, defense mechanisms, -, not in COGs.

^c^Respective gene name or symbol.

^d^Functional classification according to the KEGG (Kyoto Encyclopedia of Genes and Genomes) database.

^e^Ratio between the expression level of the *arcA* mutant versus the wild-type.

^f^A putative ArcA binding motif has been identified (+) or was not identified (-) using the Regulatory Sequence Analysis Tools (RSAT) database.

^g^Ratio between the expression level of the *fnr* mutant versus the wild-type.

^h^A putative FNR binding motif has been identified (+) or was not identified (-) using the Regulatory Sequence Analysis Tools (RSAT) database.
